# EGFR-mediated HSP70 phosphorylation facilitates PCNA association with chromatin and DNA replication

**DOI:** 10.1093/nar/gkae938

**Published:** 2024-10-29

**Authors:** Yingying Wang, Anthony Fernandez, Xinyu Pei, Bing Liu, Lei Shen, Yao Yan, Hitendra S Solanki, Lin Yang, Mian Zhou, Yuming Guo, Jun Wu, Karen L Reckamp, Li Zheng, Binghui Shen

**Affiliations:** Department of Cancer Genetics and Epigenetics, Beckman Research Institute, City of Hope, 1500 East Duarte Road, Duarte, CA 91010, USA; Department of Cancer Genetics and Epigenetics, Beckman Research Institute, City of Hope, 1500 East Duarte Road, Duarte, CA 91010, USA; Department of Cancer Genetics and Epigenetics, Beckman Research Institute, City of Hope, 1500 East Duarte Road, Duarte, CA 91010, USA; Department of Cancer Genetics and Epigenetics, Beckman Research Institute, City of Hope, 1500 East Duarte Road, Duarte, CA 91010, USA; CSL Sequirus, 225 Wyman St., Waltham, MA 02451, USA; Department of Cancer Genetics and Epigenetics, Beckman Research Institute, City of Hope, 1500 East Duarte Road, Duarte, CA 91010, USA; Department of Cancer Genetics and Epigenetics, Beckman Research Institute, City of Hope, 1500 East Duarte Road, Duarte, CA 91010, USA; Department of Cancer Genetics and Epigenetics, Beckman Research Institute, City of Hope, 1500 East Duarte Road, Duarte, CA 91010, USA; Department of Thoracic Oncology, H. Lee Moffitt Cancer Center and Research Institute, 12902 USF Magnolia Drive, Tampa, FL 33612, USA; Department of Cancer Genetics and Epigenetics, Beckman Research Institute, City of Hope, 1500 East Duarte Road, Duarte, CA 91010, USA; State Key Laboratory of Natural Medicines, School of Basic Medicine and Clinical Pharmacy, China Pharmaceutical University, 24 Tongjia Ln, Gulou, Nanjing, Jiangsu, China, 210009, China; Department of Cancer Genetics and Epigenetics, Beckman Research Institute, City of Hope, 1500 East Duarte Road, Duarte, CA 91010, USA; Animal Resource Center, Beckman Research Institute, City of Hope, 1500 East Duarte Road, Duarte, CA 91010, USA; Animal Resource Center, Beckman Research Institute, City of Hope, 1500 East Duarte Road, Duarte, CA 91010, USA; Department of Medicine, Cedars-Sinai Medical Center, 8700 Beverly Blvd, Los Angeles, CA 90048, USA; Department of Cancer Genetics and Epigenetics, Beckman Research Institute, City of Hope, 1500 East Duarte Road, Duarte, CA 91010, USA; Department of Cancer Genetics and Epigenetics, Beckman Research Institute, City of Hope, 1500 East Duarte Road, Duarte, CA 91010, USA

## Abstract

Efficient DNA replication requires highly coordinated programs for the timely recruitment of protein complexes to DNA replication forks. Defects in this process result in replication stress, which in turn activates cell cycle checkpoints, suppresses cell proliferation and induces apoptosis. In response to persistent cell growth signals that speed up DNA replication processes, cells accelerate the recruitment of DNA replication proteins to avoid DNA replication stress. The mechanisms by which cell growth signals induce processes to facilitate the recruitment of DNA replication proteins onto the replication sites remain unclear. Here, we report that the epidermal growth factor receptor (EGFR) phosphorylates heat shock protein 70 (HSP70) for DNA replication. Such a modification promotes nuclear localization and chromatin association of HSP70, which interacts with the DNA replication coordinator, proliferating cell nuclear antigen (PCNA). HSP70 subsequently facilitates the loading of PCNA onto chromatin. Knockdown or chemical inhibition of HSP70 suppresses PCNA association with chromatin and impairs DNA synthesis and Okazaki fragment maturation, leading to replicative DNA double-strand breaks and apoptosis. Furthermore, chemical inhibition of HSP70 potentiates EGFR–tyrosine kinase inhibitor-induced tumor reduction *in vivo*. This work expands our understanding of oncogenesis-induced DNA replication processes and provides a foundation for improved treatments for EGFR-mutated lung cancer by simultaneously targeting HSP70.

## Graphical Abstract

**Graphical Abstract:**
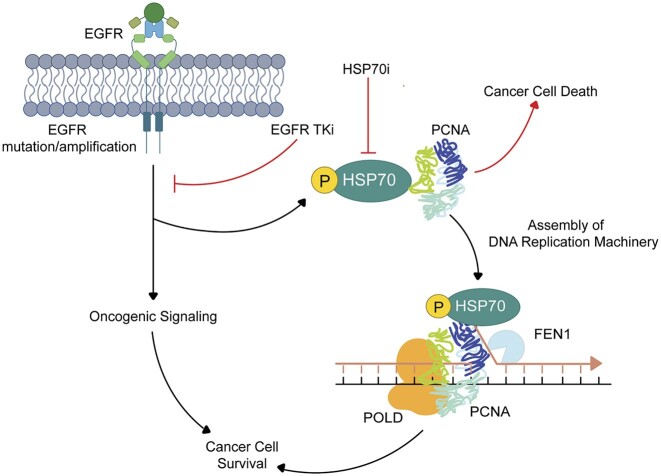

## Introduction

Efficient nuclear genomic DNA replication is fundamental to all cells. During this process, DNA polymerase epsilon catalyzes leading strand DNA synthesis in a continuous manner, and DNA polymerase delta catalyzes lagging strand DNA synthesis in the form of thousands to millions of short Okazaki fragments (1,2). Meanwhile, DNA replication accessory proteins, including proliferating cell nuclear antigen (PCNA), are required for efficient DNA synthesis (3,4). PCNA, a homotrimer complex, is loaded onto the DNA template strand and interacts with DNA polymerases to stimulate their activity and processivity (5,6). During Okazaki fragment maturation (OFM), PCNA also serves as the platform for other DNA replication components, including flap endonuclease 1 and DNA ligase 1, to effectively remove RNA primers, edit out the errors incorporated by DNA polymerase alpha and join the discrete Okazaki fragments into intact lagging strand DNA (2,7). Timely recruitment of DNA replisome enzymes and accessory proteins is crucial for efficient DNA replication. This process is closely coupled with cell proliferation. Cancer cells with aberrantly high proliferation rates have a high demand for DNA replication and require a more efficient supply and assembly of DNA replication proteins at replication forks. Deficiencies in replication proteins have been linked to replication stress, which induces cell cycle arrest and apoptosis (8,9).

Cancer cells arise due to the accumulation of genetic and/or epigenetic alterations such as those in oncogenes, including the receptor tyrosine kinases (RTKs) (10–12). Epidermal growth factor receptor (EGFR) tyrosine kinase, a major class of RTKs, is normally crucial for cell proliferation of many cell types during embryogenesis and development of adult organs (13,14). However, gene amplification and activating mutations in EGFR have been frequently observed in many human cancers, including lung adenocarcinomas (15,16). Thus, overexpression and activating mutations accelerate the TK activity of EGFR, resulting in the activation of MAPK, PI3K/AKT, mTOR and other signaling pathway activators to promote the proliferation of cancer cells (14,17). Meanwhile, activated EGFR also phosphorylates DNA replication and repair proteins, including PCNA, DNA-dependent protein kinase and histone modifying enzymes such as histone methyltransferases to regulate their functions at chromatin (18–21). We hypothesize that the wide spectrum of EGFR substrates may allow for effective coupling of cell proliferation signaling and DNA replication to avoid deficiency in replication resources.

In this work, we demonstrate that molecular chaperone heat shock protein 70 (HSP70) is both a replication fork-associated protein and a new EGFR substrate. EGFR directly phosphorylates HSP70 to maintain its stability and promote its nuclear localization. Nuclear HSP70 acts as a protein assembly factor to facilitate PCNA binding to chromatin. EGFR activation due to activating mutations and/or EGF stimulation promotes PCNA loading onto chromatin in an HSP70-dependent manner, as HSP70 knockdown or chemical inhibition suppresses chromatin association of PCNA. Consequently, HSP70 deficiency or inhibition impairs DNA replication and OFM, resulting in replicative DNA double-strand breaks (DSBs). Furthermore, HSP70 inhibition sensitizes EGFR tyrosine kinase inhibitors (TKIs) in killing EGFR-mutated HCC827 cancer cells *in vivo* and *in vitro*. The work reveals an important process in cancer cells to facilitate aberrant DNA replication and provides a new foundation for targeting HSP70 in cancer therapeutics.

## Materials and methods

### Construction of HCC827 stably expressing WT or mutant HSP70

pcDNA3.0-Flag-HSP70 wild type (HSP70-WT) was constructed according to standard subcloning protocols. In brief, the polymerase chain reaction (PCR) DNA fragment encoding Flag-tagged human HSP70 was inserted into the pcDNA3.0 vector (Addgene). The primers used for subcloning are listed in Supplementary Table S1. HSP70 mutants including pcDNA3.0-Flag-HSP70-Y41F (HSP70-Y41F, in which tyrosine-41 was mutated to nonphosphorylable phenylalanine) and pcDNA3.0-Flag-HSP70-Y41D (HSP70-Y41D, in which tyrosine-41 was mutated to phosphomimetic aspartate) were constructed using site-directed mutagenesis. All constructs were confirmed by DNA sequencing. HCC827 (*EGFR* mutant, del19, ATCC CRL-2868) cells were transfected with the pcDNA3.0-HSP70-WT, pcDNA3.0-HSP70-Y41F or pcDNA3.0-HSP70-Y41D plasmid using PolyJet™ (SignaGen) transfection reagent for 48 h. The cells were cultured in medium containing 500 μg/ml G418. Insertion of the DNA fragment encoding Flag-HSP70 into the genome was confirmed by PCR and DNA sequencing, using genomic DNA isolated from G418-resistant clones as the template. Expression of exogenous HSP70 was further confirmed by immunoblotting with an anti-Flag-tag antibody (Sigma–Aldrich).

### Cell culture and small interfering RNA transfection

HCC827 and HCC827 cells stably expressing Flag-tagged HSP70 were cultured in RPMI 1640 medium with 10% fetal bovine serum (FBS) and 1% penicillin–streptomycin antibiotics (Thermo Fisher Scientific). A549, MEF and 3T3 cells were cultured in Dulbecco’s modified Eagle’s medium medium with 10% FBS and 1% penicillin–streptomycin antibiotics (Thermo Fisher Scientific). For knockdown of HSP70 or EGFR by human HSP70 small interfering RNA (siRNA) (ORIGENE, Catalog No.: SR320559) or human EGFR siRNA (ORIGENE, Catalog No.: SR320009) transfection, cells were seeded to 50–70% confluency at the time of transfection. Lipofectamine™ 3000 reagent (Invitrogen) was diluted in Opti-MEM™ medium in a ratio of 3:100 and thoroughly mixed. Human HSP70 or EGFR siRNA was diluted in Opti-MEM™ medium and mixed with Lipofectamine™ 3000 reagent at a 1:1 ratio. The resulting DNA–lipid complex was incubated at room temperature for 15 min before being applied to the cells for transfection.

### 6× His-tag HSP70 expression and purification

pET-28b-HSP70-WT and pET-28b-HSP70-Y41F were generated by insertion of the PCR fragment encoding HSP70 (WT or Y41F) into the pET-28b vector. pET-28b-HSP70-WT and pET-28b-HSP70-Y41F plasmids were transformed into *Escherichia coli* BL21 (DE3)-competent cells (New England Biolabs). 6× His-tagged HSP70 proteins were expressed with 1 mM isopropyl-β-d-thiogalactopyranoside induction at 37°C for 4 h and purified according to the published protocol for purification of 6× His-tag HSP70 proteins (22) with modifications. Briefly, cell pellets were lysed in buffer [50 mM Tris–HCl (pH 7.5), 500 mM NaCl, 5 mM imidazole, 1 mg/ml lysozyme, 1 mM phenylmethylsulfonyl fluoride (PMSF), protease inhibitor cocktails (Sigma–Aldrich)] on ice for 30 min, followed by sonication. The supernatant was collected by centrifugation at 11 000× *g* for 10 min at 4°C. Ni-nitrilotriacetic acid resins (Clontech) were added to the supernatant. Incubation was carried out at 4°C overnight with rotation. The resins were washed three times with wash buffer [50 mM Tris–HCl (pH 7.5), 500 mM NaCl, 20 mM imidazole, 1 mM PMSF] and eluted with eluting buffer [50 mM Tris–HCl (pH 7.5), 500 mM NaCl, 200 mM imidazole, 1 mM PMSF].

### Liquid chromatography/mass spectrometry

Total cell lysates extracted from HCC827 cells, which were treated with 100 nM erlotinib or gefitinib for 72 h, were run on a 1.5 cm 4–20% Mini-PROTEAN® TGX™ precast protein gel (Bio-Rad) and stained with Coomassie brilliant blue (Thermo Fisher Scientific). The protein bands were de-stained and excised, followed by in-gel digestion using Trypsin/Lys-C Mix (Promega), according to the manufacturer’s instructions. After overnight digestion, the peptides were extracted by adding 50% ACN/0.1% TFA solution, 60% ACN/0.1% TFA solution and 80% ACN/0.1% TFA solution to the gel pieces three times. The combined peptide extracts were evaporated using a Savant SpeedVac SVC 100H centrifugal evaporator. The peptides were then dissolved in 1% formic acid (Fisher Chemical) and analyzed by reversed-phase liquid chromatography/mass spectrometry. The mass spectrometric analysis was carried out using a Thermo Scientific Orbitrap Fusion Mass Spectrometer equipped with an Easy Spray source and an Easy-nLC1000 system. The raw spectra files were searched using both Proteome Discoverer Software with Sequest (version 2.0) and the Mascot algorithm (Mascot 2.5.1).

### *In vitro* kinase assay

Active EGFR kinase domain (*N*-terminal GST-tagged human EGFR kinase domain, amino acids 696-end) was purchased from Millipore Sigma (Catalog No.: 14–531). Purified 6× His-HSP70-WT (1.5 μM) or 6× His-HSP70-Y41F (1.5 μM) and 500 nM EGFR kinase domain were added into 50 μl kinase reaction buffer [25 mM Tris–HCl (pH 7.5), 5 mM MgCl_2_, 5 mM MnCl_2_, 2 mM Dithiothreitol (DTT)] with the addition of 200 μM ATP. The reaction mixture was incubated at 37°C for 30 min and stopped by adding 6× sodium dodecyl sulphate (SDS) loading buffer. Samples were resolved with sodium dodecyl sulphate–polyacrylamide gel electrophoresis and transferred onto nitrocellulose membranes. Phosphorylated proteins were detected using an anti-phosphor-HSP70-Y41 antibody.

### Immunoprecipitation and immunoblotting

For immunoprecipitation (IP), whole cell lysates were precipitated with antibodies against specific proteins of interest and Pierce™ Protein A/G Magnetic Beads (Thermo Fisher Scientific), as we previously described (23). Briefly, cells were lysed in a buffer containing 1% Nonidet *P*-40, 50 mM Tris–HCl, 0.1 mM ethylenediaminetetraacetic acid (EDTA), 150 mM NaCl and proteinase inhibitors. The IP and corresponding input samples were analyzed by western blot. Primary antibodies used in this study are listed in Supplementary Table S2.

### Isolation of proteins on nascent DNA

Isolation of proteins on nascent DNA (iPOND) in HCC827 cells was carried out following the published protocol with slight modifications (24). Briefly, HCC827 cells were unlabeled or labeled with 5-ethynyl-2′-deoxyuridine (EdU; 10 μM, 20 min). The cells were fixed in 1% formaldehyde (10 min), and the cross-linking reaction was quenched with 0.125 M glycine solution (10 min) and washed with phosphate-buffered saline (PBS) buffer. After permeabilization, the click chemistry reaction was carried out on the fixed cells with Biotin-azide to cross-link biotin to EdU (room temperature, 60 min) following the standard click chemistry reaction protocol. The cells were then washed with PBS buffer, harvested and lysed in a lysis buffer containing 1% SDS and proteinase inhibitors in 50 mM Tris–HCl (pH 8.0). After centrifugation, the supernatant was filtered through a 90-µm nylon mesh. The clear cell lysate was then incubated with Dynabeads™ MyOne™ Streptavidin C1 (room temperature, 1 h). After washing following a previously specified washing protocol (24), the proteins were eluted with SDS sample buffer (95°C, 30 min). The resulting pulled-down proteins were analyzed by western blot analysis.

### Immunofluorescence

HCC827 cells were seeded at a density of 2× 10^5^ cells per well in 12-well plates with pre-placed coverslips and cultured in RPMI 1640 complete medium overnight. Subsequently, the cells were treated with EGF, HSP70 inhibitor VER155008 (25,26), or proteasome inhibitor MG132 (27) at the concentrations and durations specified in the figure legends. Following drug treatments, the cells were fixed in ice-cold methanol for 30 min at −20°C and then in ice-cold acetone for 15 s. The coverslips were incubated in Image-iT™ FX signal enhancer (Invitrogen) for 30 min at room temperature, followed by incubation with primary antibodies diluted in PBS at a ratio of 1:100 for 2 h at room temperature. After washing with PBS, Alexa-488-conjugated and Alexa-555-conjugated secondary antibodies (Thermo Fisher Scientific) were applied for 1.5 h at room temperature. Nuclei were stained with DAPI (Sigma–Aldrich). Microscope images were captured using fluorescence microscopy (ZEISS Axio Observer).

### Subcellular fractions and chromatin-bound protein isolation

To isolate cytoplasmic extracts (CEs) and nuclear extracts (NEs), cells were collected and suspended in CE buffer [10 mM HEPES–KOH (pH 7.5), 60 mM KCl, 1 mM DTT, 1 mM EDTA, 0.075% Nonidet *P*-40, 1× protease inhibitor cocktail] for 10 min on ice. After centrifugation (1000 × *g*, 10 min), the supernatant (CE) was collected. The pellet was then washed with CE buffer without Nonidet *P*-40 twice and resuspended in NE buffer (25% glycerol, 20 mM Tris–HCl, 420 mM NaCl, 1.5 mM MgCl_2_, 0.2 mM EDTA, 1× protease inhibitor cocktail) for 1 h on ice with intermittent vortexing. After centrifugation (20 000 × *g*, 15 min), the supernatant (NE) was collected and subjected to western blot analysis, biochemical assays or stored at −80°C.

To fractionate cytoplasmic, soluble nuclear and chromatin-bound proteins, the cells were lysed in five volumes of ice-cold Buffer A [50 mM HEPES–KOH (pH 7.5), 140 mM NaCl, 1 mM EDTA (pH 8.0), 10% glycerol, 0.5% Nonidet *P*-40, 0.25% Triton X-100, 1 mM DTT, 1× protease inhibitor cocktail]. After centrifugation (700 × *g*, 10 min), the supernatant was collected as the cytoplasm fraction and pellets were washed with Buffer A and resuspended in ice-cold Buffer B [10 mM Tris–HCl (pH 8.0), 200 mM NaCl, 1mM EDTA (pH 8.0), 0.5 mM EGTA (pH 8.0), 1× protease inhibitor cocktail]. After extraction and centrifugation (20 000 × *g*, 10 min), the soluble NE was collected. The pellet was washed with Buffer B and resuspended in Buffer C [500 mM Tris–HCl (pH 6.8), 500 mM NaCl, 1 × protease inhibitor cocktail]. The suspension was sonicated and centrifuged (20 000 × *g*, 10 min), and the resulting supernatant (chromatin-bound proteins) was collected.

### OFM assays

NE-based α-segment error editing (AEE) and RNA primer removal (RPR) assays were carried out following a previously published protocol (28). One microgram NE was incubated with oligo-based RPR or AEE DNA substrates for specific time intervals in the reaction buffer [50 mM HEPES–KOH (pH 7.5), 45 mM KCl, 5 mM MgCl_2_, 1 mM DTT, 0.1 mM EDTA, 2 mM ATP, 2 units of creatine phosphokinase, 0.5 mM NAD, 5 mM phosphocreatine] containing 5 μCi [α-^32^P] dTTP and 50 μM each of the other three dNTPs, or [α-^32^P] dATP and 50 μM each of the other three dNTPs. AEE or RPR products were resolved in 12% denaturing polyacrylamide gel electrophoresis (PAGE) and visualized by autoradiography.

### DNA fiber assay-based replication analysis

HCC827 cells were first subjected to sequential pulse labeling with 30 μM IdU for 1 h. Then, the cells were washed with fresh medium and incubated in medium containing 30 μM CIdU and either (i) Dimethyl sulfoxide (DMSO), (ii) VER155008 (10 μM), (iii) osimertinib (EGFR–TKI) (1 μM), (iv) a combination of VER155008 and osimertinib or (v) EGF (100 ng/ml) for an additional 1 h. After pulse labeling, the cells were harvested and embedded in agarose plugs (TopVision, Thermo Fisher Scientific). These cells were then lysed at 50°C in a solution containing 1% n-lauroylsarcosine, 0.5 M EDTA (pH 8.0), and 0.2 mg/ml proteinase K for 72 h. Subsequently, the lysed cells were washed with TE 8 buffer, treated with 200 μM PMSF and then washed again with TE 8 buffer before being stored in a modified TE buffer [10 mM Tris (pH 8.0) and 50 mM EDTA]. The agarose plugs were melted, and the agarose was digested using β-Agarase (New England Biolabs). The DNA was stretched on microscope slides coated with 3-aminopropyltriethoxysilane (Sigma–Aldrich). The stretched DNA was denatured in an alkali buffer [0.1 M NaOH in 70% ethanol, 0.1% β-mercaptoethanol] and fixed with 0.5% glutaraldehyde. Subsequently, the slides were blocked with 1% bovine serum albumin for 20 min. IdU and CIdU were detected using a mouse anti-IdU monoclonal antibody (BD) (1:100) and a rat anti-CIdU monoclonal antibody (Abcam) (1:500), respectively, followed by Alexa Fluor 568-conjugated goat anti-mouse (Invitrogen) (1:100) and Dylight 488-conjugated goat anti-rat (1:50) antibodies. DNA fibers were examined using fluorescence microscopy (ZEISS Axio Observer).

### Comet assay

Comet assays were carried out to analyze DNA damage using the CometAssay^®^ Kit (R&D Systems) and according to a published protocol (29). Briefly, HCC827 cells were seeded in six-well plates at a density of 2 × 10^5^ cells/well with RPMI 1640 complete medium overnight and then treated with DMSO, VER155008 (10 μM), osimertinib (5 μM) or a combination of VER155008 and osimertinib. After treatment for 24 h, cells were collected in PBS and combined at 1 × 10^5^ cells/ml with molten LMAgarose (37°C) at a ratio of 1:10 (v/v), followed by the immediate pipetting of 50 μl onto CometSlides^TM^. The slides were placed at 4°C in the dark for 30 min and then immersed in lysis buffer at 4°C overnight. After draining excessive buffer, the slides were immersed in 50 ml of 4°C 1× neutral electrophoresis buffer for 30 min, and electrophoresis was carried out (21 v, 45 min). The slides were then immersed in DNA precipitation solution [6.7 ml 7.5 M NH_4_Ac, 43.3 ml 95% ethanol] for 30 min at room temperature, followed by immersion in 70% ethanol for 30 min at room temperature. After drying at 37°C for 15 min, 100 μl of diluted SYBR™ Gold (Invitrogen) was added to each circle of dried agarose and stained for 30 min. Slides were viewed by a fluorescence microscopy (ZEISS Axio Observer).

### Apoptosis assay

HCC827 cells were seeded at a density of 2 × 10^5^ cells per well in six-well plates containing RPMI 1640 complete medium and incubated overnight. The following day, the cells were treated with DMSO, VER155008 (10 μM), osimertinib (5 μM) or a combination of VER155008 and osimertinib for 24 h. After treatment, the cells were collected, washed twice with cold PBS and resuspended in 1× binding buffer at a concentration of 1 × 10^6^ cells/ml. A 100 μl aliquot of this cell suspension (equivalent to 1 × 10^5^ cells) was transferred to a 1.5 ml tube. Apoptotic cells in each group were analyzed using the BD Pharmingen™ FITC Annexin V Apoptosis Detection Kit I and according to the manufacturer’s instructions. In brief, FITC Annexin V (5 μl) and PI (5 μl) were added to each tube. After incubation at room temperature in the dark for 15 min, 400 μl of 1× binding buffer was added to each tube, and the samples were analyzed by flow cytometry.

### Clonogenic assay

HCC827 cells were seeded at a density of 800 cells per well in 12-well plates containing RPMI 1640 complete medium and incubated overnight. The cells were treated with varying concentrations of osimertinib and/or VER155008 for 2 weeks. Colonies were then stained with crystal violet, and the area of the stained colonies was quantified by Image J.

### Tumor growth/reduction in xenograft mice

Animal studies were conducted in accordance with an approved protocol adhering to the IACUC policies and procedures at the City of Hope. Six to eight-week-old male or female NSG (NOD SCID gamma) mice were grafted with 4 million HCC827 cells per mouse. Treatment regimens were started once the tumor volume reached 400–500 mm^3^. Osimertinib and VER155008 were administered daily via gavage or intraperitoneal injection, respectively. The tumor volume in each mouse was measured twice a week and calculated using the formula of (length × width × width)/2 (mm^3^) (30). When the tumors reached a volume of 1000 mm^3^, the mice were euthanized.

### Statistical analyses

Data are shown as the mean ± SEM. The comparisons were carried out using a two-tailed Student’s *t*-test, one-way analysis of variance (ANOVA) followed by multiple comparisons with the Tukey adjustment, two-way ANOVA followed by multiple comparisons with the Tukey adjustment or Kaplan–Meier Analysis using GraphPad Prism.

## Results

### EGFR inhibition impairs DNA replication and down-regulates replication fork-associated molecular chaperones including HSP70

**Figure 1.**
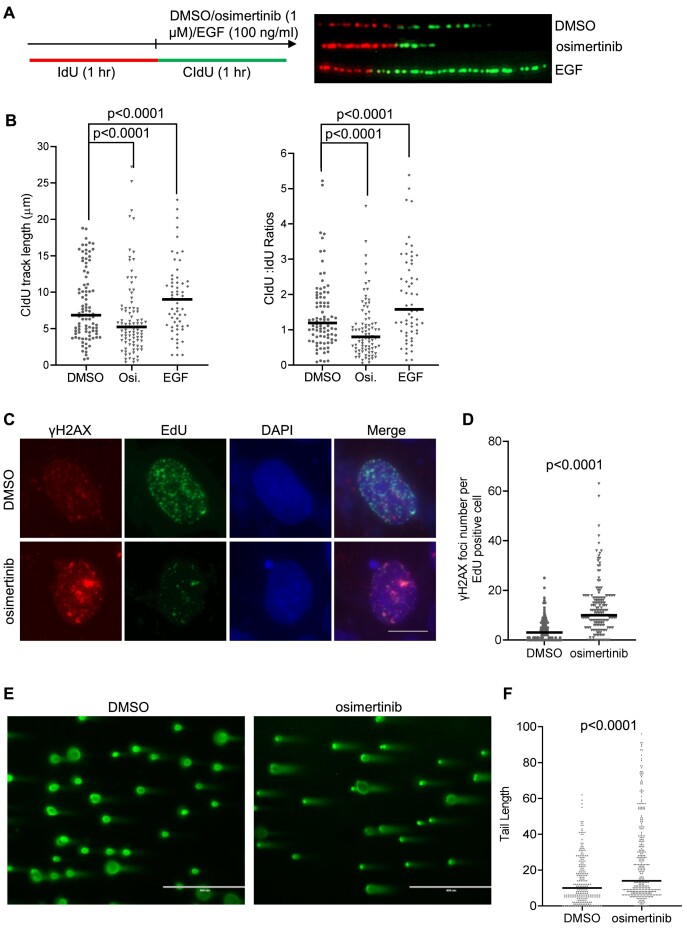
EGFR–TKI osimertinib suppresses DNA replication and induces replicative DNA damage accompanying down-regulation of chaperones. (**A**) and (**B**) Analysis of DNA synthesis with the DNA fiber assay in HCC827 cells treated with DMSO (control), osimertinib (1 μM) or EGF (100 ng/ml) for 1 h. Panel (A) shows the diagram of IdU and CIdU labeling and the drug treatment (upper panel) and representative fiber images in the control, osimertinib and EGF-treated cells. Panel (B) shows the CIdU track length in the IdU labeled tracks (left panel) and the CldU/IdU ratio (right panel). IdU and CIdU tracks were scored and the ratio was calculated in each sample using the Image J software. (**C**) and (**D**) γH2AX foci in replicating cells untreated or treated with osimertinib (5 μM) for 2 h. Panel (C) shows the representative image of γH2AX foci in EdU-positive cells. HCC827 cells were pre-treated with DMSO (untreated control) or osimertinib and labeled with EdU (10 μM) for 30 min. The EdU-positive cells were stained with the Click-reaction, and γH2AX foci were stained with the antibody against γH2AX. Scale bar: 20 μm. Panel (D) shows quantification of γH2AX foci number in the EdU-positive cells using the Image J program. (**E**) and (**F**) Genome wide DNA damage analysis with the comet assay in HCC827 cells treated with DMSO (control) or osimertinib (5 μM) for 24 h. Panel (E) shows the representative images of nuclei and panel (F) is the quantification of comet tail in the control and osimertinib-treated cells. *P*-values were calculated using the Student’s *t*-test.

We have proposed that EGFR activation induces mechanisms to facilitate DNA replication and cell proliferation. If this is true, then inhibition of EGFR will block the mechanisms, leading to replication stress. To test this hypothesis, we treated HCC827 cells with the selective, third-generation EGFR TKI, osimertinib (31), and evaluated its impact on DNA replication. We observed that treatment with osimertinib significantly reduced DNA synthesis (Figure 1A and B). At the same time, we treated HCC827 cells with EGF and found that EGFR activation by EGF facilitates DNA replication (Figure 1A and B). Consistently, osimertinib-treated or EGFR-depleted cells had significantly higher levels of γH2AX foci in the EdU-positive cells than the untreated control cells (Figure 1C and D, and Supplementary Figure S1A–C), indicating that the osimertinib causes replicative DNA strand breaks. Comet assays showed that osimertinib-treated cells had significantly more DNA damage than the untreated control cells (Figure 1E and F).

EGFR inhibition has been shown to reduce PCNA levels (32). To determine if EGFR inhibition causes reduction of DNA replication proteins, we used mass spectrometry-based proteomics to estimate global protein levels. We compared the list of proteins significantly down-regulated by EGFR–TKIs, erlotinib or gefitinib (33) with previously reported DNA replication fork-associated proteins defined by the iPOND procedure (24). Of the 321 EGFR–TKI down-regulated proteins, 60 proteins were associated with replication forks. Pathway analysis revealed that chaperone proteins including heat shock protein HSP70, which is encoded by the *HSPA1A* gene, were among the most enriched clusters of proteins (Supplementary Table S3). This family of molecular chaperones was previously reported to mediate protein degradation and protein complex assembly (34–36). Using western blot analysis, we confirmed that osimertinib or erlotinib reduced HSP70 protein levels (Supplementary Figure S2A). iPOND western blot analysis further showed that HSP70 is associated with DNA replication forks in HCC827 cells (Supplementary Figure S2B). On the other hand, our iPOND, which did not include the thymidine chase control, did not define whether HSP70 preferentially bind to replication forks. These observations suggested that HSP70 is an EGFR-mediated DNA replication protein.

### HSP70 is an EGFR tyrosine kinase substrate

**Figure 2.**
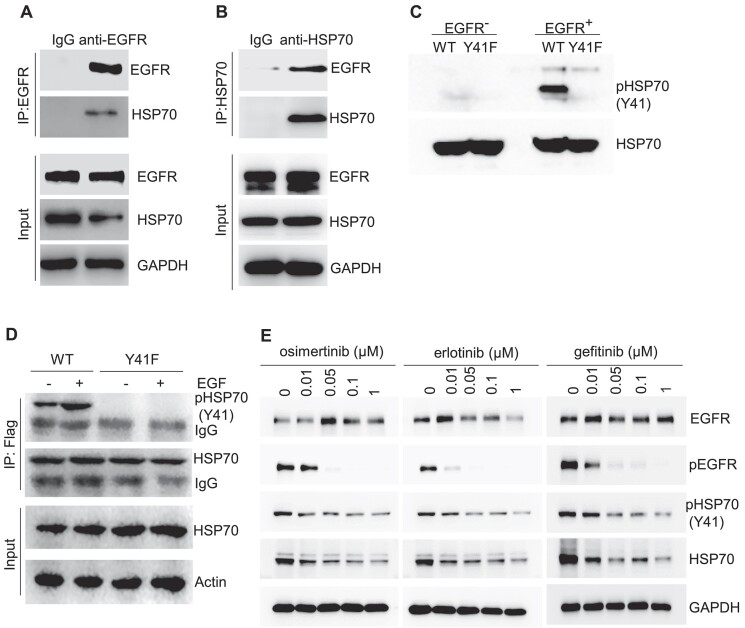
EGFR interacts and phosphorylates HSP70. (**A**) and (**B**) HSP70–EGFR interaction in HCC827 cells. The HSP70–EGFR complex was immunoprecipitated from HCC827 cell lysates using antibodies against HSP70 (panel A) or EGFR (panel B), respectively. The immunoprecipitated HSP70–EGFR complex was analyzed by western blot. (**C**) *In vitro* EGFR kinase assay detecting Y41 phosphorylation of HSP70. Purified EGFR kinase domain protein was used as the enzyme and purified Flag-tagged HSP70 (WT or Y41F) was used as the protein substrate. HSP70 Y41 phosphorylation was evaluated by western blot using the antibody against Y41 phosphorylated HSP70. (**D**) Enhancement of HSP70 phosphorylation by EGF. HCC827 cells expressing Flag-tagged WT or Y41F HSP70 were untreated or treated with EGF (100 ng/ml, 1 h). The phosphorylation of Flag-tagged HSP70 (WT or Y41F) was analyzed by Co-IP using anti-Flag-tag antibody and western blot using the antibody against Y41 phosphorylated HSP70. (**E**) Inhibition of HSP70 phosphorylation by EGFR–TKIs. HCC827 cells were treated with EGFR–TKIs osimertinib, erlotinib and gefitinib. The status of EGFR phosphorylation, EGFR level, HSP70 phosphorylation and HSP70 level were analyzed by western blot. GADPH was used as the loading control for western blot.

HSP70, which we previously showed to be destabilized by erlotinib or gefitinib (37), was among the replication fork-associated heat shock proteins. We confirmed that osimertinib reduced HSP70 protein levels in HCC827 cells (Supplementary Figure S3A and B). While we previously demonstrated that EGFR inhibition prevented its phosphorylation at Y41 residue (37), it is not clear whether the phosphorylation is a direct result of EGFR kinase activity. To test if HSP70 is a substrate of EGFR tyrosine kinase, we performed co-immunoprecipitation (Co-IP) experiments in *EGFR* E19del HCC827 cells. HSP70 and EGFR were pulled down reciprocally with specific antibodies (Figure 2A and B), suggesting a physical binding of HSP70 to EGFR. We further conducted an *in vitro* EGFR tyrosine kinase activity assay using purified EGFR as enzyme and purified recombinant WT or Y41F mutant HSP70 as substrates. EGFR effectively phosphorylated WT HSP70 but not the Y41F mutant (Figure 2C). We observed that HSP70 was phosphorylated in HCC827 cells, and EGF stimulation enhanced HSP70 phosphorylation (Figure 2D). However, EGFR inhibition by erlotinib, gefitinib or osimertinib reduced HSP70 Y41 phosphorylation (Figure 2E). These findings demonstrate that HSP70 is a direct substrate of EGFR tyrosine kinase.

### EGFR-mediated phosphorylation promotes HSP70 nuclear translocation

**Figure 3.**
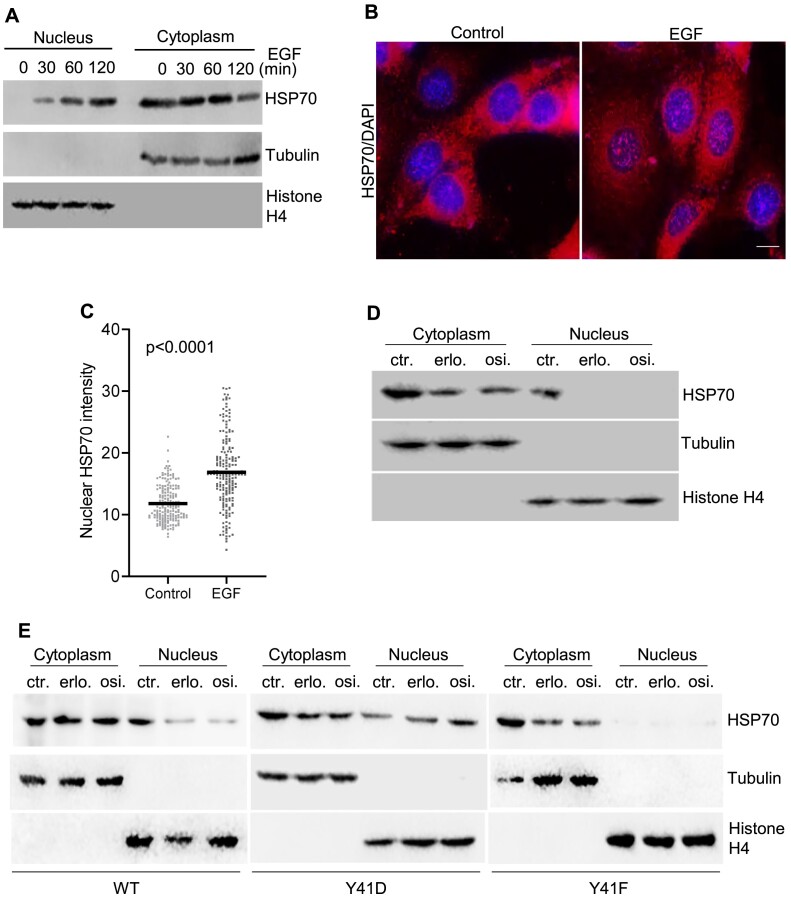
EGFR mediated Y41 phosphorylation of HSP70 promotes HSP70 nuclear translocation. (A–C) Stimulation of HSP70 nuclear localization by EGF. (**A**) HCC827 cells were treated with EGF (100 ng/ml) for 0, 30, 60 and 120 min. Cytoplasmic and nuclear HSP70 was analyzed by western blot. (**B**) and (**C**) HCC827 cells were untreated or treated with EGF (100 ng/ml) for 30 min. Immunofluorescence (IF) staining of HSP70 was carried out to evaluate HSP70 in the nucleus (DAPI stained). Panel (B) shows the representative IF images with or without EGF treatment. Panel (C) is the quantification of nuclear HSP70 by Image J. The *P*-value was calculated using Student’s *t*-test. Scale bar: 20 μm. (**D**) EGFR–TKIs block HSP70 nuclear localization. HCC827 cells were untreated or treated with erlotinib (erlo.) (1 μM) or osimertinib (osi.) (1 μM) for 24 h. Cytoplasmic and nuclear HSP70 was analyzed by western blot. (**E**) Impact of HSP70 phosphorylation on its nuclear localization. HCC827 cells expressing WT, phosphorylation mimicking Y41D and phosphorylation-deficient mimicking Flag-tagged HSP70 were treated with erlotinib (erlo.) (1 μM) or osimertinib (osi.) (1 μM) for 24 h. Cytoplasmic and nuclear Flag-tagged WT, Y41D or Y41F HSP70 was analyzed by western blot.

Next, we investigated the impact of EGFR-mediated HSP70 phosphorylation on its nuclear localization. Western blot analysis revealed that under normal culture conditions, there was no detectable nuclear fraction of HSP70 in HCC827 cells (Figure 3A). However, EGF stimulation of HCC827 cells for 0, 30, 60 and 120 min greatly increased the level of nuclear HSP70 in a time-dependent manner (Figure 3A). Consistent with western blot analyses, immunofluorescence staining of HSP70 showed that EGF stimulation promoted HSP70 nuclear localization (Figure 3B and C). A similar phenomenon was observed in A549 and 3T3 cells as well (Supplementary Figure S4A–D). These results suggest that EGFR-mediated HSP70 nuclear localization is a common phenomenon in mammalian cells. Conversely, treatment of HCC827 cells with osimertinib reduced the total pool and nuclear HSP70 levels (Figure 3D, and Supplementary Figure S2A). To further address if EGFR-mediated Y41 phosphorylation is crucial for HSP70 nuclear localization, we analyzed nuclear localization of WT HSP70, phosphorylation-mimicking Y41D HSP70 and phosphorylation-deficient Y41F HSP70 in HCC827 cells. We found that treatment of the cells with erlotinib or osimertinib to block EGFR-mediated HSP70 phosphorylation reduced the nuclear accumulation of WT HSP70, but there was no change in the phosphorylation-mimicking Y41D HSP70 (Figure 3E, and Supplementary Figure S5). We observed minimal nuclear Y41F mutant protein, regardless of EGFR–TKI treatment (Figure 3E, and Supplementary Figure S5). On the other hand, we found that EGFR–TKIs could still cause HSP70 degradation (Supplementary Figure S5). It suggests additional mechanism mediating HSP70 stability. These results suggest that EGFR-mediated HSP70 Y41 phosphorylation is required for nuclear translocation of HSP70, in addition to maintenance of its stability.

### EGFR-mediated HSP70 Y41 phosphorylation facilitates PCNA binding to chromatin

**Figure 4.**
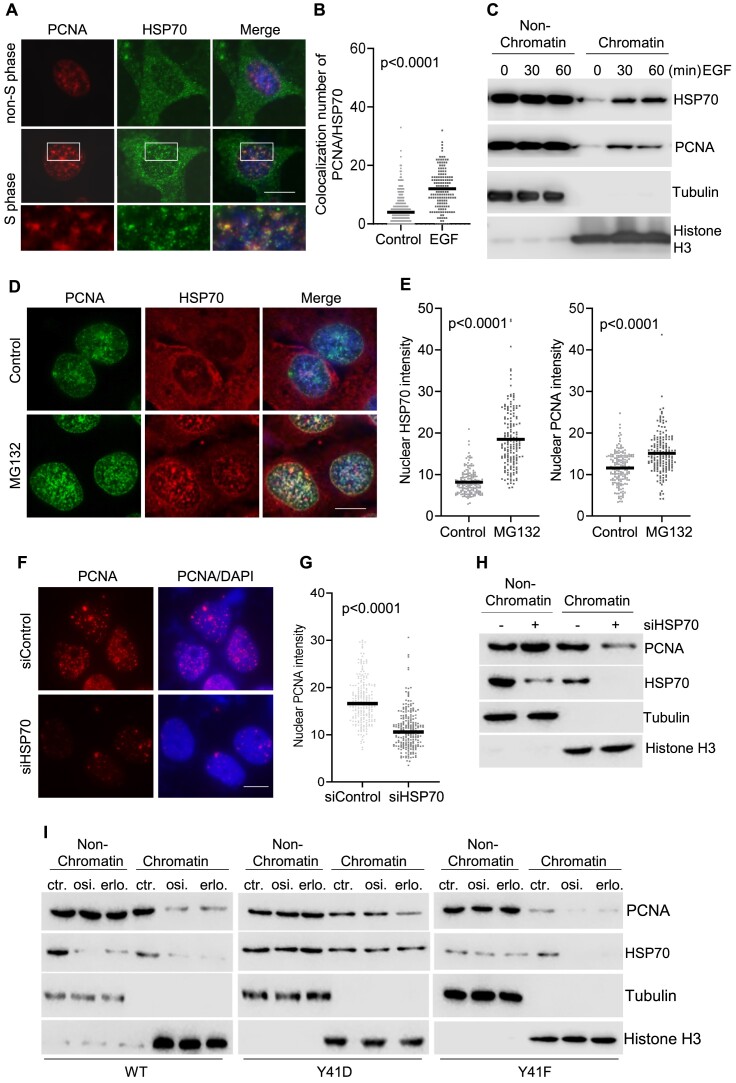
EGFR-mediated HSP70 phosphorylation facilitates PCNA association with chromatin. (**A**) HSP70 and PCNA were co-stained using specific antibodies against these two proteins. Nuclei were stained with DAPI. Representative images of interphase (with few PCNA foci) and S phase (with clear PCNA foci) HCC827 cells were shown. (**B**) Quantification of HSP70 foci that were co-localized with PCNA foci in HCC827 cells untreated or treated with EGF (100 ng/ml, 30 min). (**C**) EGF stimulated chromatin association of HSP70 and PCNA. Nonchromatin and chromatin fractions were prepared from HCC827 cells treated with EGF (100 ng/ml) for 0, 30 and 60 min. HSP70 and PCNA in these fractions were analyzed by western blot. Tubulin and histone H3 were used as the loading controls for nonchromatin and chromatin fractions, respectively. (**D**) and (**E**) Impact of MG132 on the level of nuclear HSP70 and PCNA. Panel (D) shows representative co-IF staining images of HSP70 and PCNA in HCC827 cells without or with MG132 treatment (1 μM, 1 h) and panel (E) is the quantification of nuclear HSP70 and PCNA in the cells as shown in panel (D). (**F**) and (**G**) IF staining of PCNA in parental and HSP70 knockdown cells. HCC827 cells HSP70 knockdown was performed by siRNA transfection. Panel (F) shows the representative image and panel (G) is the quantification. (**H**) Impact of HSP70 knockdown on chromatin association of PCNA. PCNA expression from each cell fraction was examined by western blotting. Tubulin and histone H3 were used as the loading controls for nonchromatin and chromatin fractions, respectively. (**I**) Impact of HSP70 phosphorylation on chromatin association of PCNA. HCC827 cells expressing WT, phosphorylation mimicking Y41D and phosphorylation-deficient mimicking Y41 Flag-tagged HSP70 were untreated (ctr.) or treated with erlotinib (erlo.) (1 μM) or osimertinib (osi.) (1 μM) for 24 h. Nonchromatin and chromatin fractions were prepared from these cells. The level of nonchromatin PCNA and chromatin associated PCNA and HSP70 were analyzed by western blot. Tubulin and histone H3 were used as loading controls for nonchromatin and chromatin fraction, respectively. The *P*-values in panels (B), (E) and (G) were calculated using Student’s *t*-test.

PCNA is a key regulatory protein that acts as a scaffold to recruit DNA replication and repair proteins onto chromatin (3,38). Recruitment of PCNA is crucial for efficient DNA replication (5). Our previous proteomics analysis of PCNA binding proteins indicated that HSP70 binds to PCNA in response to DNA base damage (37). To determine the role of nuclear HSP70 in DNA replication, we co-stained HSP70 and PCNA. We observed that HSP70 co-localized with PCNA in S phase cells, which had clear PCNA foci, but not in the non-S phase cells (Figure 4A). EGF enhanced nuclear HSP70 and PCNA foci (Figure 4B). In addition, we observed that phosphorylation-mimicking Y41D, but not phosphorylation-deficient Y41F HSP70 mutation enhanced nuclear HSP70 foci, which co-localized with PCNA foci (Supplementary Figure S6A and B). We further found that EGF considerably enhanced the chromatin association of both HSP70 and PCNA (Figure 4C). In addition, proteasome inhibitor MG132, which blocks osimertinib-induced HSP70 degradation (Supplementary Figure S7), increased nuclear HSP70 and was accompanied by an increase in PCNA foci intensity (Figure 4D and E).

To determine if HSP70 enhances PCNA loading onto chromatin, we knocked down HSP70 in HCC827 cells (Supplementary Figure S8). We found that the depletion of HSP70 decreased the level of chromatin-bound PCNA (Figure 4F–H). At the same time, we found that the HSP70 inhibitor VER155008 significantly reduced PCNA loading onto chromatin (Supplementary Figure S9A). Inhibition of HSP70 by VER155008 also moderately reduced the loading of MCM proteins and POLD1 onto chromatin, but it did not affect RFC loading onto chromatin (Supplementary Figure S9A). Consistently, IF staining showed that HSP70 inhibitor VER155008 significantly suppressed PCNA foci formation in HCC827 cells when treated with EGF (Supplementary Figure S9B and C). On the other hand, ATM inhibitor AZD1390 only moderately inhibited PCNA association with chromatin, and AZD1390 did not restore PCNA loading onto chromatin in the presence of osimertinib (Supplementary Figure S10). To determine if EGFR-mediated HSP70 Y41 phosphorylation is important for HSP70-assisted PCNA chromatin loading, we treated HCC827 cells expressing WT or phosphorylation-deficient Y41F HSP70 with EGF. EGF induced PCNA chromatin association in the HCC827 cells expressing WT but not Y41F HSP70 (Supplementary Figure S11A). In contrast, HCC827 cells stably expressing Y41D HSP70 had a higher level of chromatin associated PCNA (Supplementary Figure S11B) under normal culture conditions. Next, we treated HCC827 cells expressing the WT, Y41D or Y41F HSP70 with EGFR–TKIs erlotinib or osimertinib to block HSP70 phosphorylation. We found that erlotinib or osimertinib inhibited chromatin association of both WT HSP70 and PCNA (Figure 4I). On the other hand, the expression of phosphorylation-mimicking Y41D HSP70 but not Y41F HSP70 partially restored PCNA loading onto chromatin in the presence of EGFR–TKIs (Figure 4I). These findings suggest that EGFR-mediated HSP70 phosphorylation is crucial for promoting PCNA loading onto chromatin.

### HSP70 promotes OFM and replication fork progression

**Figure 5.**
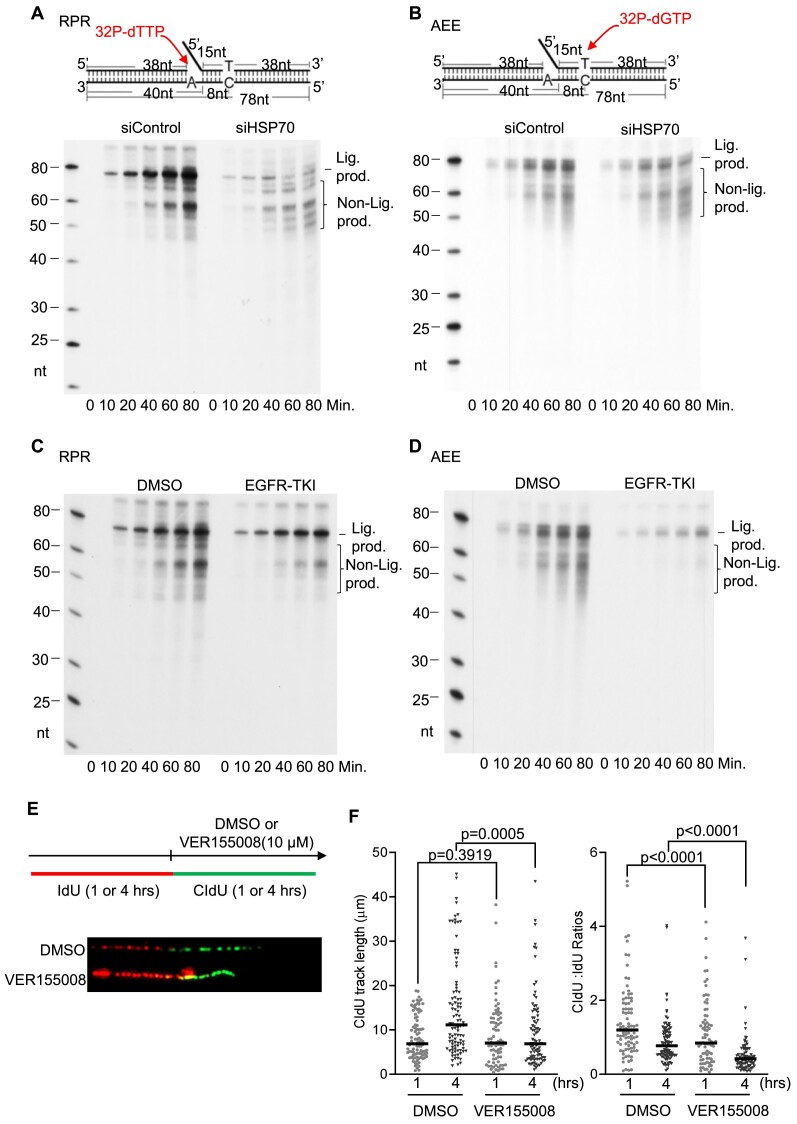
HSP70 deficiency or chemical inhibition impairs OFM and DNA replication. (**A**–**D**) NEs-based OFM assays. Panels (A) and (C) show RPR assays using the NEs isolated from parental or HSP70 knockdown HCC827 cells or cells treated with EGFR–TKI (erlotinib, 1 μM) for 24 h, and panels (B) and (D) show the Polα error editing (AEE) assays using NEs isolated from parental or HSP70 knockdown cells or cells treated with EGFR–TKI (erlotinib, 1 μM) for 24 h. The diagrams on the top of panels (A) and (B) are the oligo-based DNA substrates. The images in panels (A–D) are the representative denaturing PAGE image of the RPR and AEE reaction products. Reactions were carried out at 37°C for 0, 10, 20, 40, 60 and 80 min. The unligated products (Nonlig. prod.) and ligated (Lig. prod.) were indicated. (**E**) and (**F**) Analysis of DNA synthesis with the DNA fiber assays in HCC827 cells treated with DMSO (control, 1 or 4 h) or HSP70 inhibitor VER155008 (1 or 4 h). Panel (E) shows the diagram of IdU and CldU labeling and the drug treatment (upper portion) and representative fiber images in the control and VER155008-treated cells (bottom portion). Panel (F) shows the CldU track length in the IdU labeled tracks. The *P*-values in panels (F) were calculated using Student’s *t*-test.

PCNA is required for efficient DNA synthesis and the completion of OFM during DNA replication. Given that HSP70 promotes PCNA binding to chromatin for its functions, HSP70 deficiency should impair DNA synthesis and OFM. To test this hypothesis, we knocked down HSP70 and assayed RPR and Polα error editing reactions (AEE), which are two key processes during OFM (7,28), using NEs. We observed that HSP70 knockdown remarkably reduced RPR and AEE efficiency (Figure 5A and B). Similar to HSP70 knockdown, RPR and AEE assays using the NEs from the HCC827 cells treated with erlotinib showed a remarkable reduction of OFM reaction efficiency compared with the untreated cells (Figure 5C and D). DNA fiber analysis on DNA synthesis in HCC827 cells revealed that inhibition of HSP70 by its selective inhibitor VER155008 significantly suppressed replication fork extension (Figure 5E and F). In addition, EGF, which stimulates HSP70 phosphorylation and HSP70-assisted PCNA chromatin association, promoted DNA replication in the WT but not Y41F HCC827 cells (Supplementary Figure S12A and B). On the other hand, Y41D HCC827 cells had a significantly higher DNA replication rate than the WT (Supplementary Figure S12C and D). It supports the idea that HSP70 promotes DNA synthesis, and its deficiency impairs DNA replication due to a lack of sufficient PCNA.

### Pharmacological inhibition of the EGFR–HSP70 signaling axis results in replication-related DNA damage and cell apoptosis

**Figure 6.**
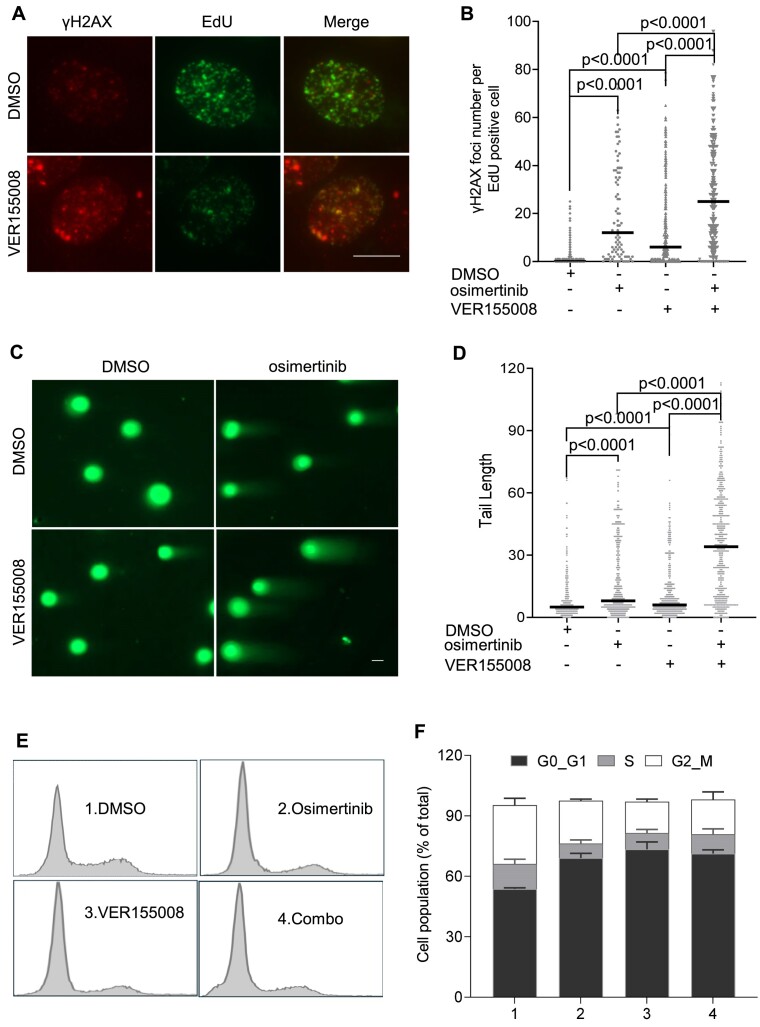
HSP70 inhibition causes replicative DNA damage and alters cell cycle progression. (**A**) and (**B**) γH2AX foci in replicating untreated cells or cells treated with HSP70 inhibitor VER155008 (5 μM) and osimertinib (5 μM) individually or in combination for 2 h. Panel (A) shows the representative image of γH2AX foci in EdU-positive cells. HCC827 cells were pre-treated with DMSO (untreated control) or VER155008 (5 μM) for 2 h. The cells were labeled with EdU (10 μM) for 30 min. The γH2AX-EdU co-staining was carried out and γH2AX foci in EdU positive cells were scored as described in Figure 1. Scale bar: 20 μm. Panel (B) shows quantification of γH2AX foci number in the EdU-positive cells that were pre-treated with DMSO (untreated control) or VER155008 and osimertinib individually or in combination for 2 h using Image J. (**C**) and (**D**) Genome wide DNA damage analysis with the comet assays in HCC827 cells treated with DMSO (control) or VER155008 (10 μM) and osimertinib (5 μM) individually or in combination for 24 h. Panel (C) shows the representative images of nuclei in the control and treatment groups and panel (D) is the quantification of comet tails in the control or VER155008 and/or osimertinib-treated cells. (**E**) and (**F**) Flow cytometry-based cell cycle analysis of HCC827 cells treated with DMSO (untreated control) or VER155008 (10 μM) and osimertinib (5 μM) individually or in combination for 24 h. Panel (E) shows the PI-stained cells in each group and panel (F) indicated the percentages of each cell cycle phase in different groups of HCC827 cells [(i) DMSO, (ii) osimertinib (5 μM), (iii) VER155008 (10 μM) and (iv) combination of osimertinib and VER155008) for 24 h. The *P*-values in panels (B) and (D) were calculated using Student’s *t*-test.

To assess whether blocking HSP70, similarly to osimertinib, causes replication-related DNA strand breaks in HCC827 cells, we evaluated γH2AX foci formation in replicating (EdU-positive) HCC827 cells. Under normal culture conditions, only a few EdU-positive cells showed γH2AX foci. However, pre-treatment of the cells with osimertinib or VER155008 alone or in combination for 2 h significantly increased the number of cells with γH2AX foci (Figure 6A and B). The mean γH2AX foci number in osimertinib or VER155008-treated cells was 20 and 14, respectively, compared with <2 in the untreated control. Cells treated with a combination of osimertinib and VER155008 had an average of 29 γH2AX foci. In addition, we also observed significantly higher levels of γH2AX foci in EdU-negative HCC827 cells treated with osimertinib and/or VER155008 than in the EdU-negative untreated control cells (Supplementary Figure S13). In addition, osimertinib and VER155008 also induced DNA damage in other cell lines including 3T3 and A549 cells. We observed that osimertinib alone significantly increased γH2AX foci (Supplementary Figure S14A–D). VER155008 on its own did not increase the γH2AX level. However, combination of osimertinib with VER155008 caused more γH2AX foci than osimertinib alone in 3T3 or A549 cells (Supplementary Figure S14A–D).

We previously found that HSP70 plays a role in the base excision repair pathway (37). Consistent with previous reports, we observed that base-damaging agent methyl methane sulfonate (MMS) induced HSP70 or PCNA colocalization with XRCC1, a key BER protein that serves as a molecular marker of BER foci (Supplementary Figure S15A and B). Therefore, we sought to determine the impact of EGFR or HSP70 inhibition on the BER pathway in HCC827 cells. We found that under normal growth conditions, the signal of aldehyde reactive probe (ARP), which has been used to detect base damage in cells, was low, and EGFR or HSP70 inhibitors, unlike MMS, did not significantly affect ARP signals (Supplementary Figure S16A and B). These data suggest that HSP70 may be important in repairing base damage in response to MMS. However, under normal conditions, inhibition of EGFR or HSP70 has little impact on the accumulation of base damage-related ssDNA breaks (SSBs), which, if not repaired, may collapse DNA replication forks and DSBs. Unligated Okazaki fragments and base damage related SSBs are two major types of SSBs. Because inhibition of EGFR or HSP70 did not significantly cause base damage, the DSBs we observed in cells treated with EGFR–TKI or HSP70i are mainly resulted from unligated Okazaki fragments.

We further used the comet assay to analyze the genome-wide DSB levels of cells treated with osimertinib and/or VER155008. We found that both osimertinib and VER155008 significantly increased the average length of the comet tail. The mean length of comet tails of osimertinib- or VER155008-treated cells were 18 and 11 μm, respectively, compared to 8 μm for the untreated control (Figure 6C and D). The cells treated with both osimertinib and VER155008 showed a mean comet tail length of 36 μm. These data suggest that functional inhibition of HSP70 by VER155008, like EGFR inhibition by osimertinib, causes DSBs, including replication-related DSBs, and it strongly potentiates osimertinib to induce DSBs in HCC827 cells. In parallel to the DNA replication deficiency, HSP70 inhibition alters cell cycle progression. We observed a significant increase in the G0/G1 cell population in cells treated with osimertinib, VER155008, or their combination (Figure 6E and F).

### HSP70 inhibitor VER155008 sensitizes HCC827 cells to EGFR inhibitor osimertinib both *in vitro* and *in vivo*

**Figure 7.**
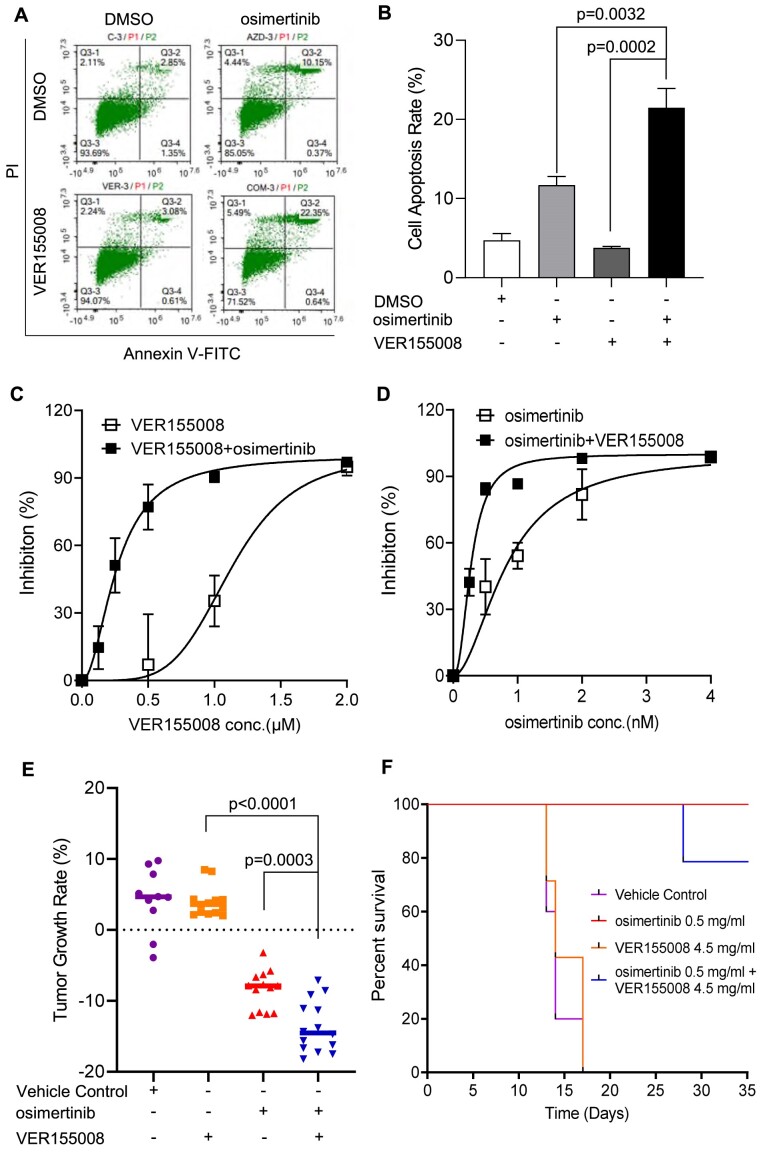
HSP70 inhibitor VER155008 and EGFR–TKI osimertinib synergistically kill HCC827 cells *in vitro* and *in vivo*. (**A**) and (**B**) Flow cytometry-based apoptosis analysis of HCC827 cells treated with DMSO (untreated control) or VER155008 (10 μM) and osimertinib (5 μM) individually or in combination for 24 h. Panel (A) shows the Annexin V-FITC and PI co-stained cells in each group and panel (B) indicated the percentage of apoptotic cells (Q2 in panel A) in different groups of HCC827 cells. (**C**) and (**D**) The synergy between the HSP70 inhibitor VER155008 and the EGFR–TKI inhibitor osimertinib was assayed by clonogenic assay. The values are means ± SEM of three independent clonogenic assays. Panel (C) is the inhibition curve of varying concentrations of VER155008 from 0 to 2 μM in combination with osimertinib (0 or 2 nM). Panel (D) is the inhibition curve of varying concentrations of osimertinib from 0 to 4 nM in combination with VER155008 (0 or 1 μM). (**E**) and (**F**) VER155008 potentiates osimertinib-induced HCC827 tumor cell killing in xenografting mice. Panel (E) shows the tumor growth rate in mice treated with DMSO (untreated control, *n* = 10) or VER155008 (*n* = 13) and osimertinib (*n* = 13) individually or in combination (*n* = 14). *P*-values were calculated using the Student's *t*-test. Panel (F) is the survival curve of each group.

We next determined if HSP70 inhibition by VER155008 affects cell proliferation and survival and whether it sensitizes osimertinib in killing HCC827 cells. Treatment with the HSP70 inhibitor at 10 μM for 24 h, unlike osimertinib, did not significantly induce cell apoptosis compared to the control solvent DMSO (Figure 7A and B). However, VER155008 strongly enhanced osimertinib-induced apoptosis (Figure 7A and B). We further carried out colony-formation assay to define the impact of HSP70 inhibition on the survival and proliferation of HCC827, cells without or with a combination of osimertinib. We treated the cells with varying concentrations of VER155008 (0–2 μM) in the absence or presence of 2 nM osimertinib. In a parallel experiment, we treated HCC827, with different concentrations of osimertinib (0–4 nM) in the absence or presence of 1 μM VER155008 for 2 weeks. The IC_50_ of VER155008 alone or osimertinib alone on clonogenicity of HCC827 cells was 1.1 μM or 0.8 nM, respectively (Figure 7C and D). In the presence of osimertinib, the IC_50_ of VER155008 was reduced to 0.3 μM, and in the presence of VER155008 the IC_50_ of osimertinib was reduced to 0.3 nM (Figure 7C, D). This suggests that VER155008 and osimertinib potentiate each other in killing HCC827 cells. In addition, we observed that VER155008 induced more apoptosis in the Y41F HCC827 cell population than in the WT (Supplementary Figure S17A–C). Consistently, the colony-forming assay also showed that Y41F cells were more sensitive to VER155008 (Supplementary Figure S17D). These findings suggest that HSP70 promotes DNA replication and cell survival through an EGFR-mediated Y41 phosphorylation mechanism as well as Y41 phosphorylation-independent mechanisms.

To evaluate this phenomenon *in vivo*, we assessed the effect of combination treatment VER155008 with osimertinib on tumor growth in HCC827 cell-xenografted mice. Mice were either untreated, treated with VER155008 alone, osimertinib alone, or a combination of VER155008 and osimertinib. Osimertinib at 1.7 mg/kg/day dose had a significant tumor reduction effect as a single agent (Figure 7E). At 15 mg/kg/day dose, we did not observe a significant tumor reduction effect by VER155008 (Figure 7E). However, VER155008 in combination with osimertinib was significantly more efficient in reducing tumor growth than the VER155008 or osimertinib alone (Figure 7E). In addition, Kaplan–Meier survival analyses showed that osimertinib alone prolonged mice survival, but osimertinib in combination with VER155008 did not show an additional beneficial effect in prolonging the survival time of mice (Figure 7F).

## Discussion

Our this study demonstrates that HSP70 plays important roles in facilitating both DNA synthesis and OFM. In response to EGFR activation, HSP70 is phosphorylated by EGFR TK and migrates into the nucleus. The nuclear HSP70 interacts with the DNA replication protein PCNA and assists PCNA to load onto chromatin. Knockdown or chemical inhibition of HSP70 impairs PCNA recruitment to chromatin and suppresses DNA synthesis and OFM. Consequently, HSP70 deficiency or inhibition causes replication-related DNA damage and cell cycle arrest.

It is worth noting that HSP70 localization to replication forks is mostly observed in transformed cells but not in normal cells (Supplementary Figure S18). It suggests that HSP70-assisted PCNA loading onto chromatin is not a canonical process for DNA replication. Instead, this process is needed only in cells under growth stimulus conditions such as exposure to EGF or other growth factors, or in cells that have undergone oncogenic transformation. In normal cells, the regular replisome assembly process is sufficient for DNA replication and cell division (39). However, in transformed cells, which are under persistent proliferative stimulation, there are high demands for DNA replication (10,11). Therefore, regular replisome assembly cannot fulfill the requirements of abnormally fast DNA replication, and additional protein factors are needed to facilitate replisome assembly (40,41). Our this study suggests that HSP70 is an important factor for the chromatin loading of PCNA. Because PCNA is the master platform for recruiting core DNA replication enzymes, including polymerases, nucleases, helicases and ligases, for different DNA transactions during replication, its accelerated loading onto chromatin speeds up the recruitment of all the key DNA replication enzymes to replication forks for their reactions (4,5).

*EGFR*-mutated NSCLC harbors oncogenic driver mutations in the *EGFR* gene (42,43). *EGFR* mutations activate EGFR and trigger a cascade of signaling pathways that lead to oncogenesis, which promotes cell proliferation (14,17). Intriguingly, due to oncogene activation, cancer-initiating cells simultaneously undergo cell cycle arrest and apoptosis due to overwhelming replication stress (44–46). To become malignant, the cancer-initiating cells must induce a mechanism to counteract the replication stress. EGFR-mediated HSP70 nuclear localization and HSP70-assisted assembly of the PCNA-centered replisome are important mechanisms for cancer-initiating cells to reduce replication stress for their proliferation. Both EGFR and HSP70 inhibition effectively suppress PCNA association with chromatin and cause DNA replication-related DNA damage and cell cycle arrest. Furthermore, HSP70 inhibitor VER155008 significantly sensitizes HCC827 cells to osimertinib-induced accumulation of DNA damage, cell cycle arrest and/or apoptosis.

HSP70 was originally identified as a homolog of the *E. coli* chaperone protein DnaK (47). As a chaperone molecule, HSP70 mediates protein folding and refolding (48). It plays an important role in stabilizing proteins and protecting its protein substrates from forming aggregates under stress conditions (49). Recent studies have shown that HSP70 also mediates protein membrane translocation and protein–protein interactions (50). HSP70 was found to be overexpressed in human cancers, and its high expression was linked to poor prognosis (51,52). Because of its function in counteracting stress conditions and its overexpression status in human cancers, HSP70 has been proposed as a target for cancer treatment. Our this study suggests a new mechanism of HSP70 inhibition to block DNA replication and induce DNA replication stress in certain types of human cancer cells, including EGFR-mutated cancer cells. This provides new insights into the actions of HSP70 inhibitors such as VER155008 in cancer treatment.

## Supplementary Material

gkae938_Supplemental_File

## Data Availability

The mass spectrometry proteomics data have been deposited to the ProteomeXchange Consortium via the PRIDE partner repository with the dataset identifier PXD050420 and 10.6019/PXD050420.
