# Supplementary material for: EGFR-mediated HSP70 phosphorylation facilitates PCNA association with chromatin and DNA replication
Source: Nucleic Acids Res. 2024 Oct 29;52(21):13057–72. doi: 10.1093/nar/gkae938 (PMC11602123; doi:10.1093/nar/gkae938)
Supplement: gkae938_Supplemental_File [file gkae938_supplemental_file.pdf]

Table S1: Primer information of DNA constructs

Primer name	Sequence
pcDNA3.0-HSP70-WT sense	CGCGGATCCGGACTACAAAGACGATGACGACAAGAT GGCCAAAGCCGCGGCGATC
pcDNA3.0-HSP70-WT antisense	AAGGAAAAAAGCGGCCGCATCTACCTCCTCAATGGTG
pcDNA3.0-HSP70-Y41D sense	ACCACCCCCAGCGACGTGGCCT
pcDNA3.0-HSP70-Y41D antisense	GAAGGCCACGTCGCTGGGGGTG
pcDNA3.0-HSP70-Y41F sense	ACCACCCCCAGCTTCGTGGCCT
pcDNA3.0-HSP70-Y41F antisense	GAAGGCCACGAAGCTGGGGGTG

Table S2: Antibody information for western blotting

Name	Vendor	Catalog no.	Dilution
Anti-HSP70	Thermo Fisher Scientific	MA3-028	1:500
Anti-HSP70-Y41P	Thermo Fisher Scientific	PA5-36042	1:500
Anti-Flag	Millipore Sigma	F1804	1:1000
Anti-PCNA	Cell Signaling Technology	13110	1:500
Anti-EGFR	Cell Signaling Technology	4267	1:1000
Anti-pEGFR	Cell Signaling Technology	2234	1:1000
Anti-GAPDH	Cell Signaling Technology	2118	1:3000
Anti-Tubulin	Santa Cruz Biotechnology	Sc-8035	1:1000
Anti-Histone H4	Santa Cruz Biotechnology	Sc-25260	1:1000
Anti-Histone H3	Cell Signaling Technology	4499	1:3000
Anti-Actin	GeneTex	GTX108639	1:3000
Anti-MCM2	Cell Signaling Technology	3619	1:1000
Anti-POLD1	Abclonal	A4218	1:1000
Anti-RFC1	Abclonal	A1625	1:1000
Anti-XRCC1	Cell Signaling Technology	76998	1:1000

Table S3: EGFR-TKI down-regulated functional clusters and corresponding proteins

Term	Fold Enrichment	P Value	Genes
ATPases(HSPA1A)	10.52	3.90E-10	CCT3, HSP90AA1, RFC3, RFC4, RFC2, DDX23, SMC1A, SMC2, PSMC3, PSMC4, CCT8, CCT7, CCT5, HSPA1A
Chaperones	15.24	1.03E-09	CCT3, HSP90AA1, DNAJC7, CDC37, AHSA1, TBCB, CCT8, RBBP7, CCT7, CCT5, HSPA1A
Replisome	38.8	3.8E-07	RFC3, RFC4, PCNA, RFC2, POLD1, CDK1
DNA repair	6.90	0.001606	RFC3, RFC4, RFC2, POLD1, CDK1, SMC1A
Glycolysis	33.42	1.28E-05	GPI, PGK1, ENO1, GAPDH, PFKP
Proteasome	25.44	4.97E-04	PSMA1, PSMC3, PSMC4, TXNL1

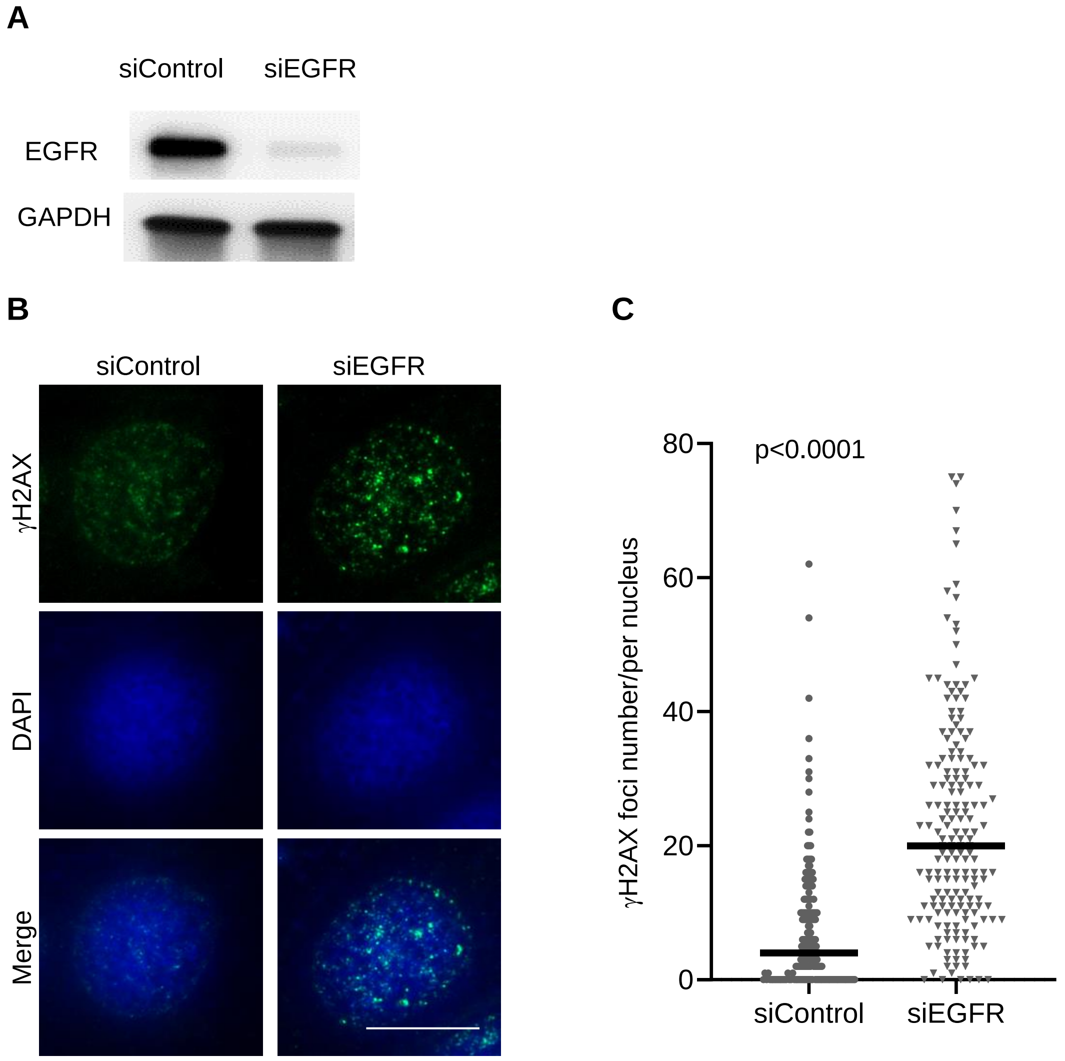

Supplementary Figure S1. Impact of EGFR knockdown on induction of DNA damage in HCC827 cells. (A) EGFR protein levels were detected in the presence of siEGFR by western blotting. (B) Representative images of immunofluorescence staining of γH2AX (green) in HCC827 cells without or with EGFR knockdown. Nuclei were counterstained with DAPI (blue). Scale bar: 20 µm. (C) Quantification of the number of γH2AX foci in HCC827 cells without or with EGFR knockdown using the Image J software. The p-value was calculated using the student's t-test.

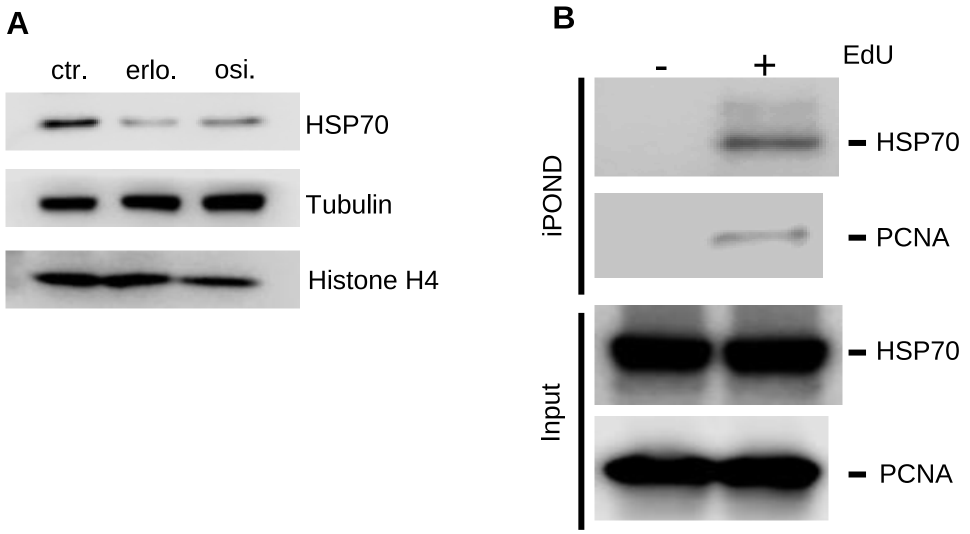

Supplementary Figure S2. HSP70 is a DNA replication protein that is mediated by EGFR. (A) Western blot analysis showing total HSP70 protein levels in whole cell lysates from HCC827 cells treated without or with EGFR TKIs erlotinib (1 µM) or osimertinib (1 µM) for 24 hours. (B) iPOND was performed on HCC827 cells. The association of HSP70 with nascent DNA strand was analyzed by western blot. PCNA was used as a positive control. HCC827 cells without EdU labeling were used as a negative control.

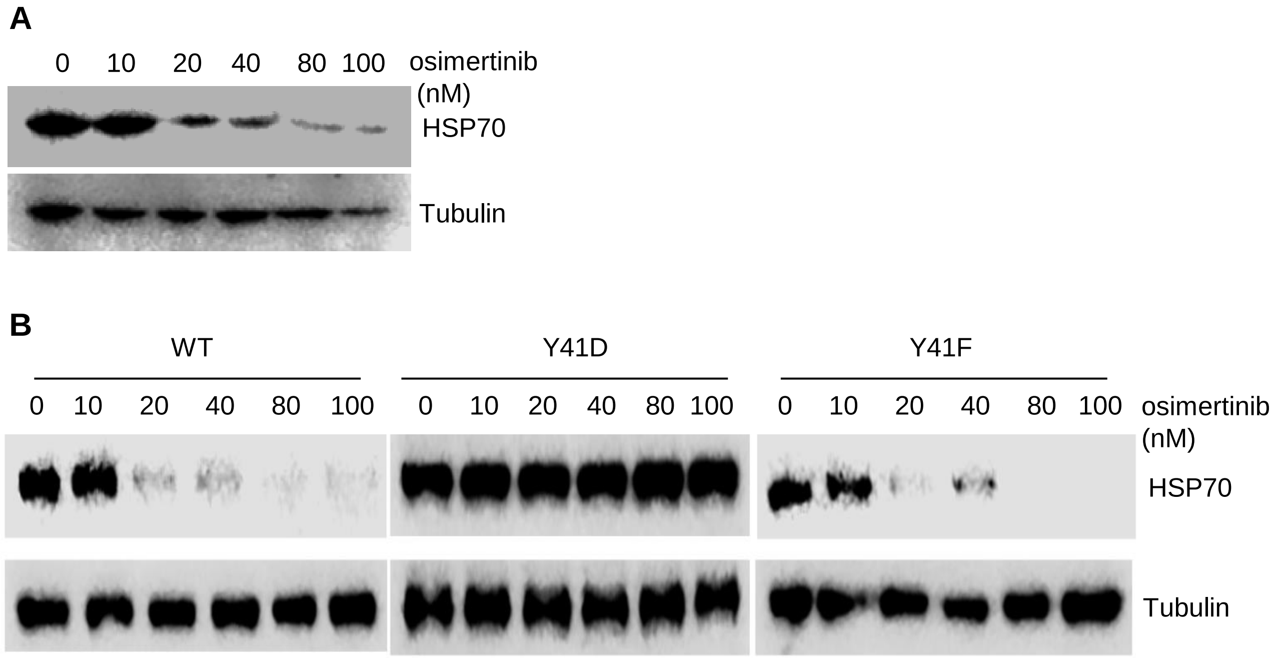

Supplementary Figure S3. Western blotting to show that the protein expression of HSP70 following EGFR TKI osimertinib treatment for 48 hours. (A) Cellular extracts were prepared from osimertinib-treated HCC827 cells as indicated. HSP70 protein presence was detected by western blotting. (B) WT, Y41D, and Y41F HSP70 protein expression were measured in the presence of the indicated concentrations of osimertinib at different concentrations from 10-100 nM by western blotting.

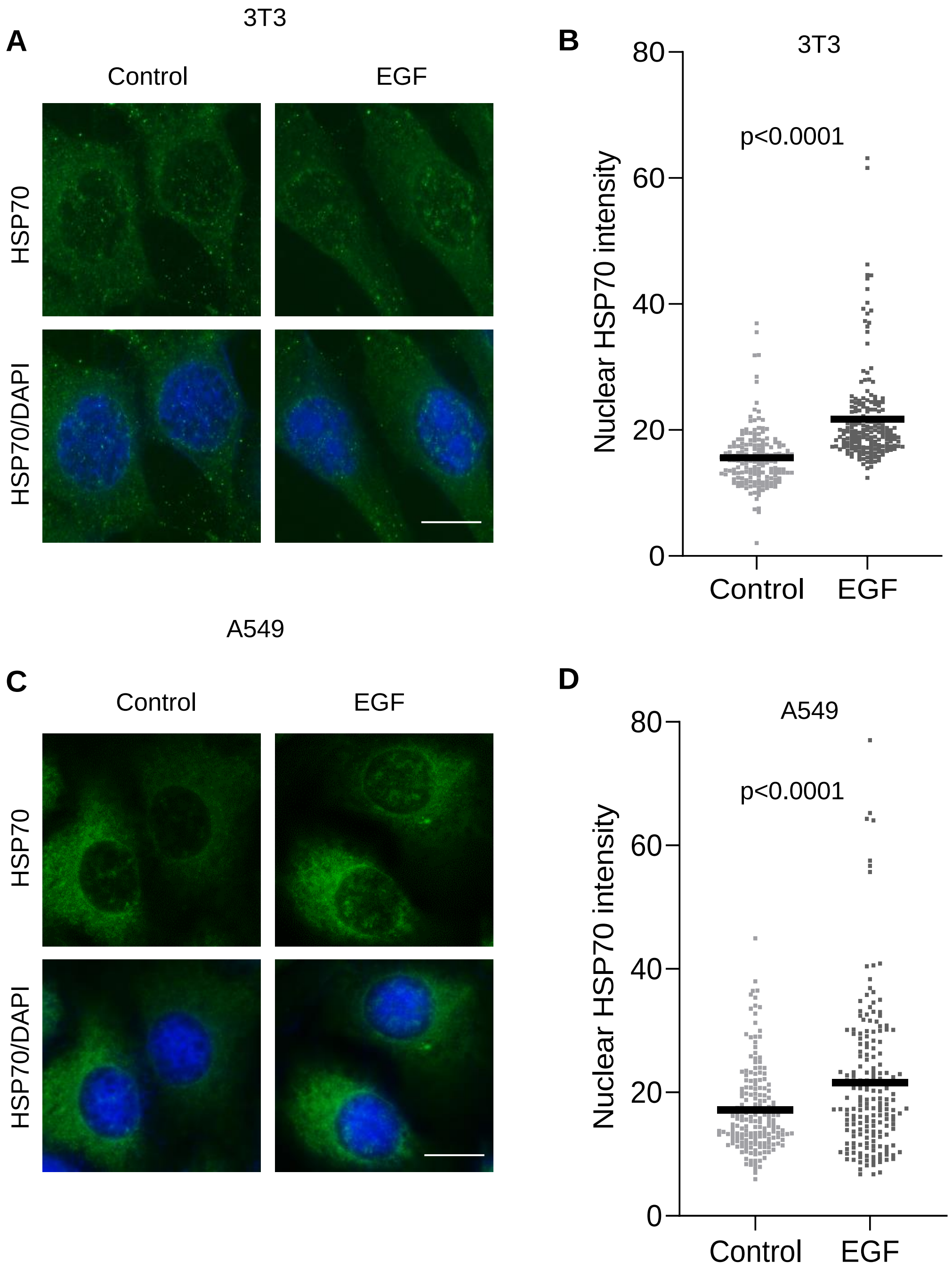

Supplementary Figure S4. Immunofluorescence staining to show that EGFR activation promotes HSP70 protein nuclear translocation. (A) and (C) Representative images of IF staining of HSP70 in 3T3 and A549 cells following treatment with or without 100 ng/ml hEGF for 1 hour. Nuclei were counterstained with DAPI (blue). Scale bar: 20 µm. (B) and (D) Quantification of nuclear HSP70 intensity using Image J software. The p-value was calculated using the student's t-test.

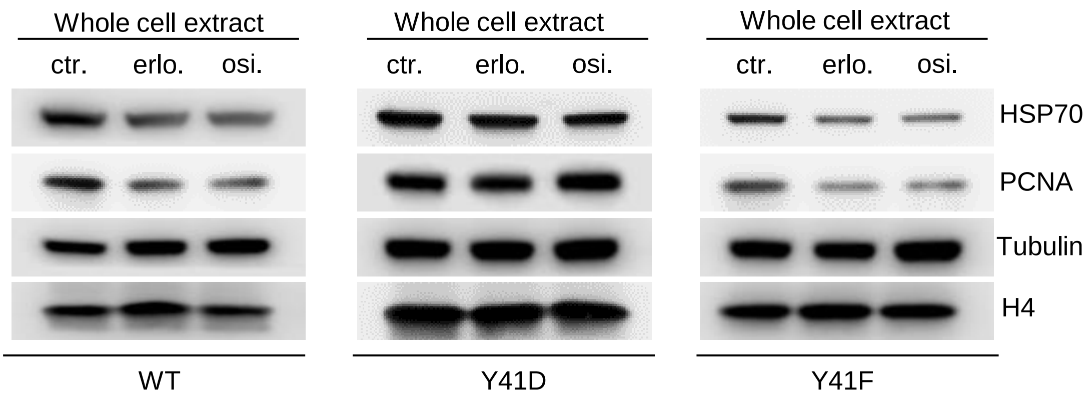

Supplementary Figure S5. Western blotting to show that the protein expression of HSP70 and PCNA following EGFR TKIs erlotinib (1 μM) or osimertinib (1 μM) treatment for 24 hours in WT HSP70, Y41D, and Y41F mutant HCC827 cells.

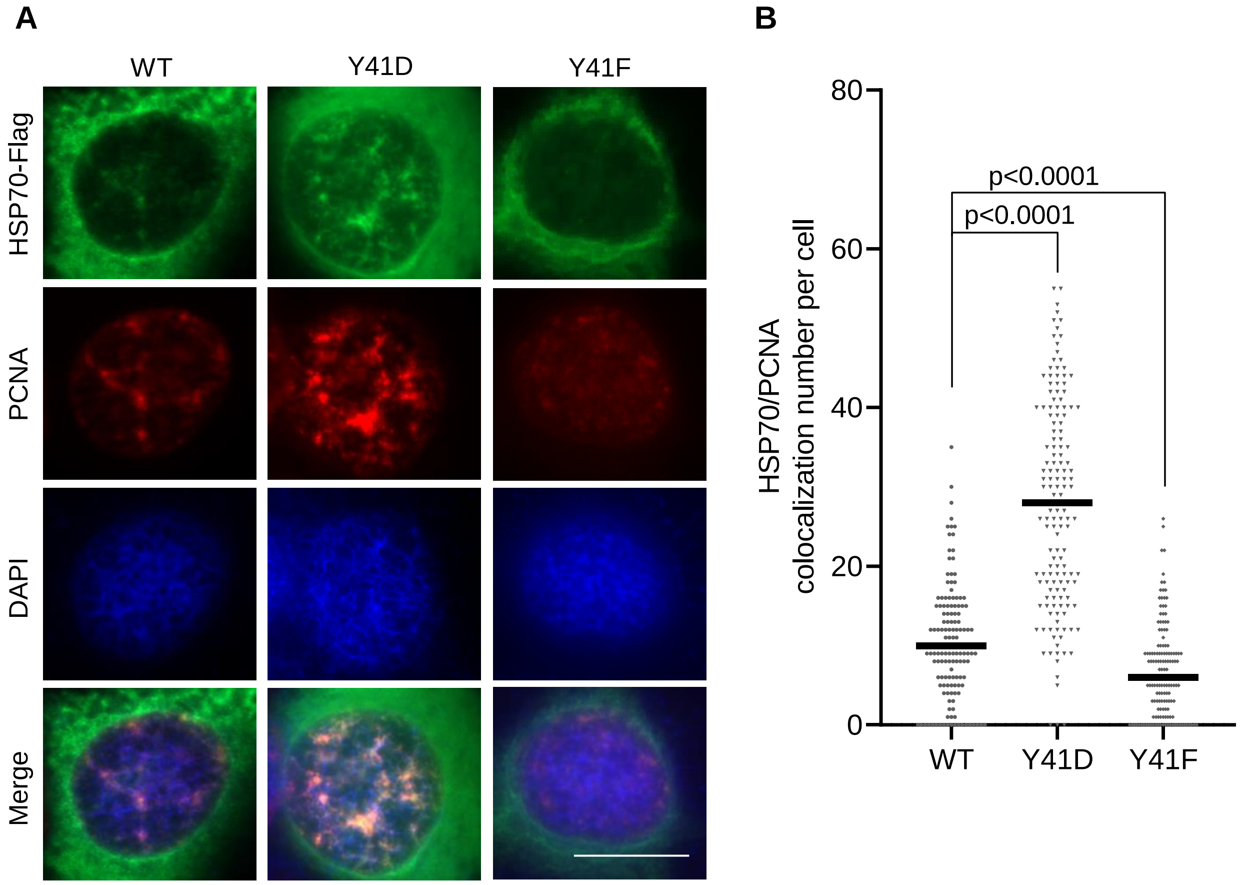

Supplementary Figure S6. Colocalization of PCNA with Flag-tagged WT, Y41D, and Y41F HSP70. (A) representative images of co-immunofluorescence staining of PCNA (red) and different Flag-tagged HSP70 proteins (green) in HCC827 cells. Nuclei were counterstained with DAPI (blue). Scale bar: 20 μm. (B) Quantification of HSP70 foci that co-localized with PCNA foci, using Image J software. The p-value was calculated using the student's t-test.

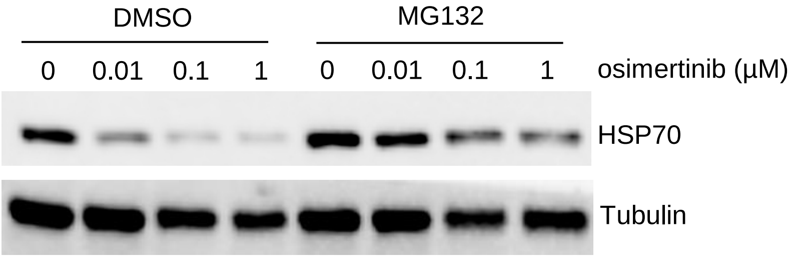

Supplementary Figure S7. Western blotting was used to detect levels of HSP70 under the treatment of osimertinib in the absence or presence of the proteasome inhibitor MG132.

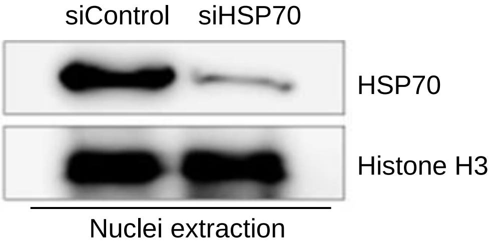

Supplementary Figure S8. Western blotting to show HSP70 protein expression from nuclear extract after HSP70 siRNA knockdown.

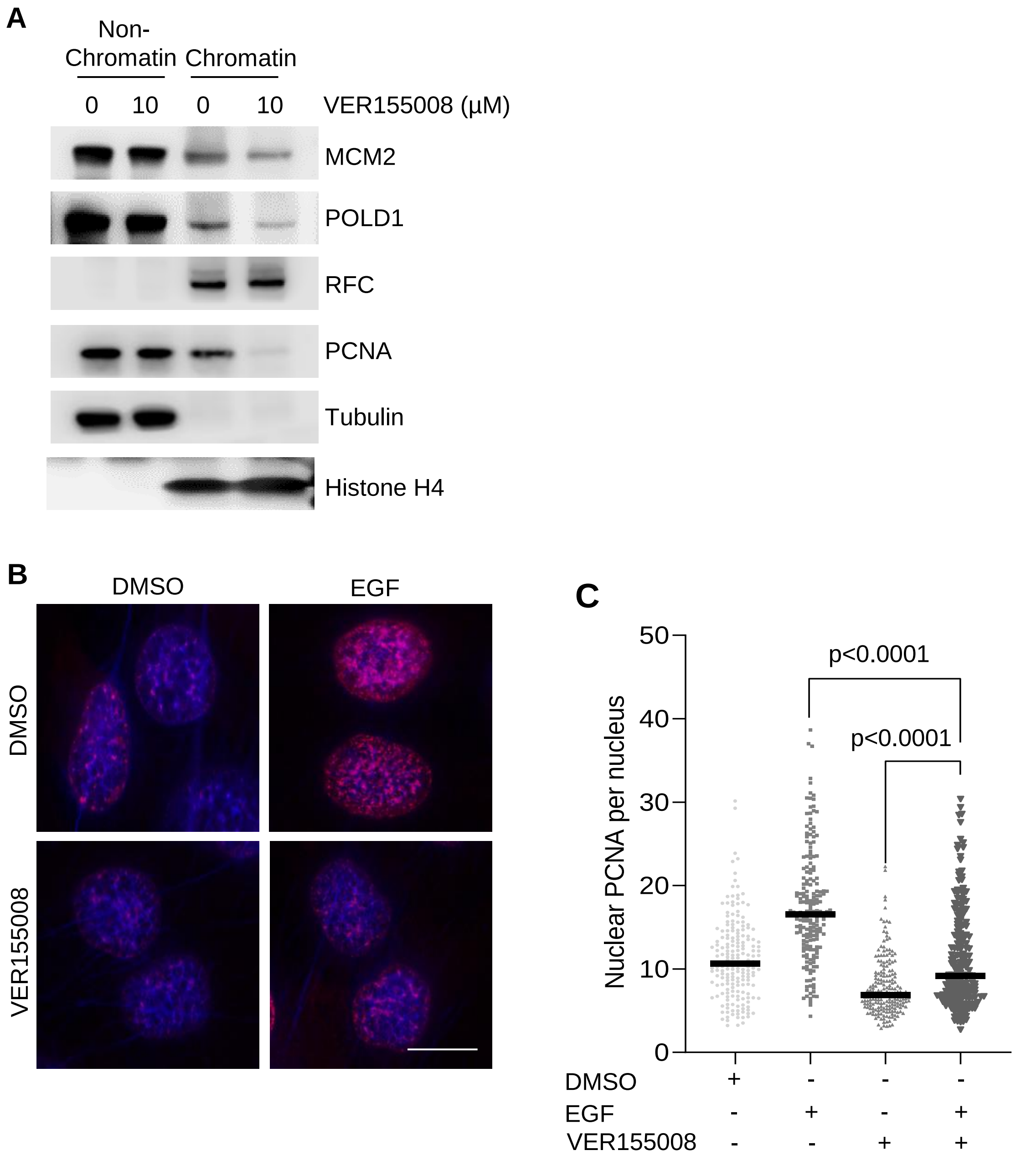

Supplementary Figure S9. HSP70 promotes PCNA binding to chromatin for its functions. (A) HCC827 cells were treated with HSP70 inhibitor VER155008 (0, 10 µM) overnight. Non-chromatin or chromatin associated PCNA, MCM2, POLD1, and RFC were examined by western blotting. Tubulin and histone H4 were used as the loading controls for non-chromatin and chromatin fractions, respectively. (B) IF staining was conducted to visualize nuclear PCNA foci in HCC827 cells treated with or without HSP70 inhibitor VER155008 (10 µM, overnight) followed with EGF exposure (100 ng/mL, 1 h). Nuclei were counterstained with DAPI. Scale bar: 20 µm. (C) Quantification of nuclear PCNA intensity. The p-value was calculated using Student's t-test.

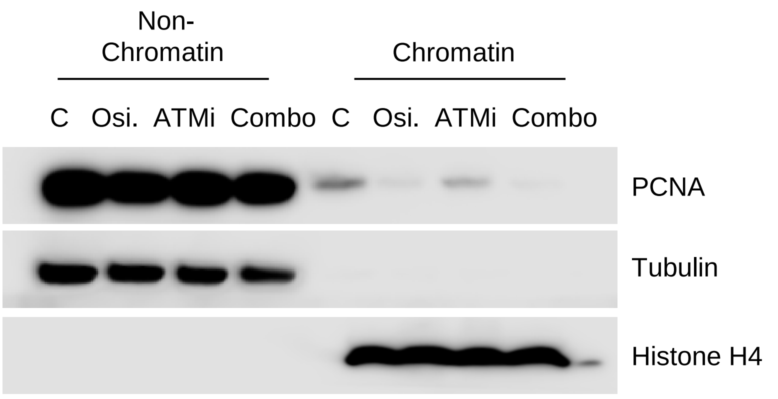

Supplementary Figure S10. Impact of ATM inhibition on chromatin association with PCNA. HCC827 cells were treated with osimertinib (100 nM) or ATM inhibitor (ATMi) AZD1390 (10 µM) overnight. Non-chromatin or chromatin associated PCNA was examined by western blotting. Tubulin and histone H4 were used as the loading controls for non-chromatin and chromatin fractions, respectively.

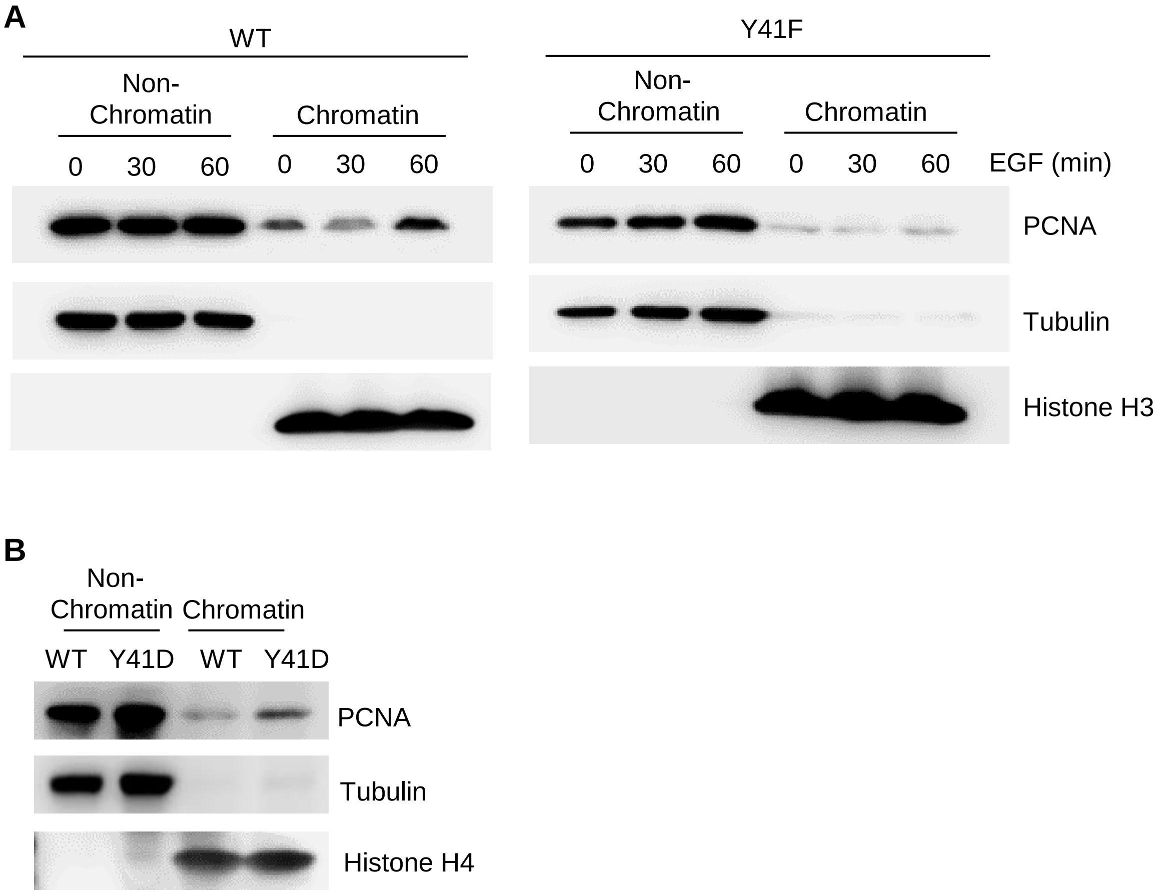

Supplementary Figure S11. Impact of phosphorylation deficient Y41F or phosphorylation mimicking Y41D HSP70 mutation on PCNA chromatin-loading. (A) HCC827 cells expressing WT or Y41F were exposed to EGF (100 ng/ml). Non-chromatin or chromatin PCNA were detected with western blotting. (B) HCC827 cells expressing WT or Y41D HSP70 were cultured at normal conditions. Non-chromatin or chromatin PCNA were detected with western blotting.

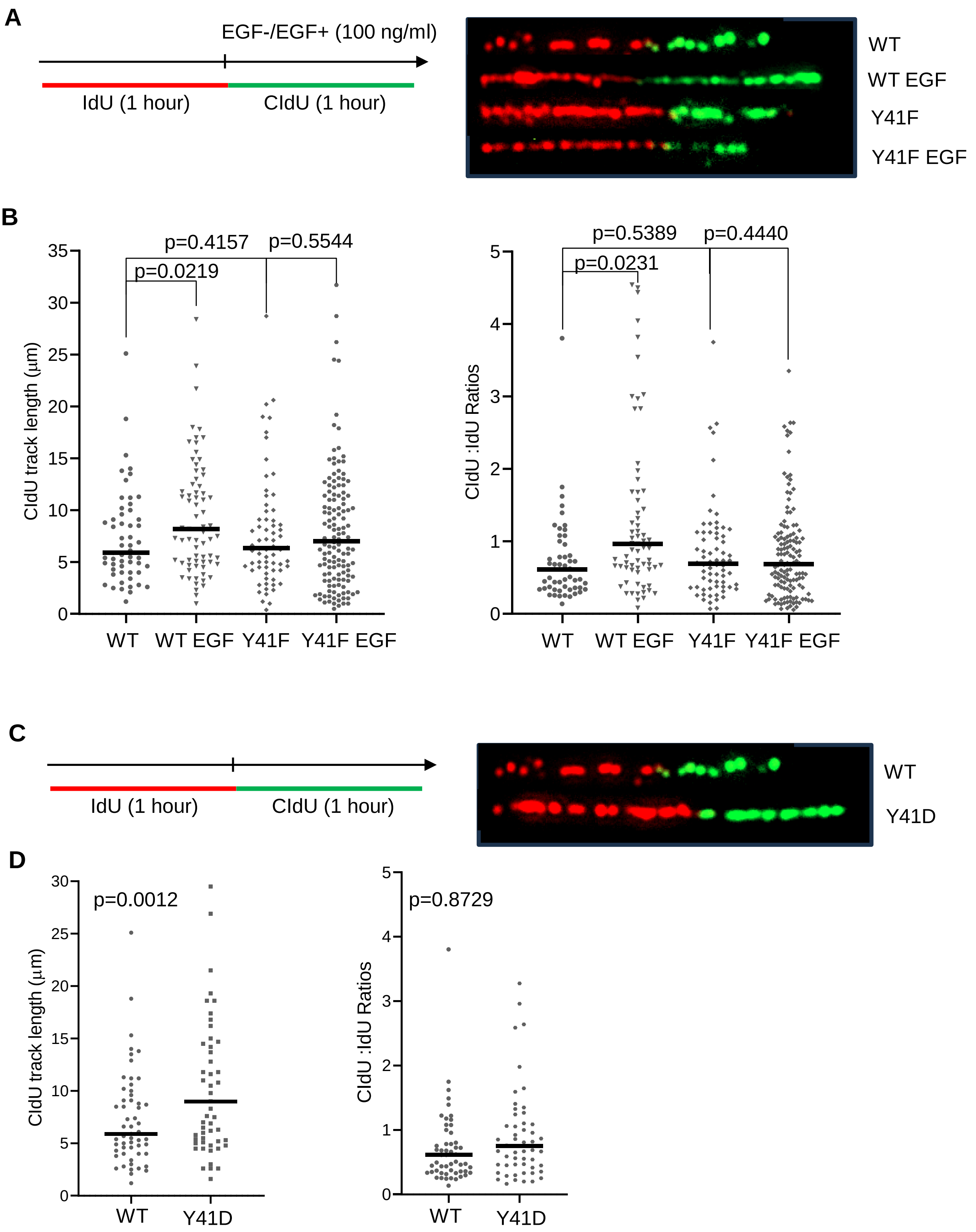

Supplementary Figure S12. Replication rates in WT, Y41F, or Y41D HCC827 cells. (A) The diagram of IdU (Red) and C1dU (Green) labeling and EGF treatment (upper panel) and representative fiber images in EGF untreated or treated WT and Y41F HCC827 cells. (B) C1dU track length in IdU labeled tracks and the C1dU/IdU ratio in EGF untreated or treated WT and Y41F HCC827 cells. (C) The diagram of IdU (Red) and C1dU (Green) labeling and representative fiber images in WT and Y41D HCC827 cells. (D) C1dU track length in IdU labeled tracks and the C1dU/IdU ratio in WT or Y41D HCC827 cells under the normal growth condition. IdU and C1dU (Green) tracks were scored and the ratio was calculated in each sample using the Image J software.

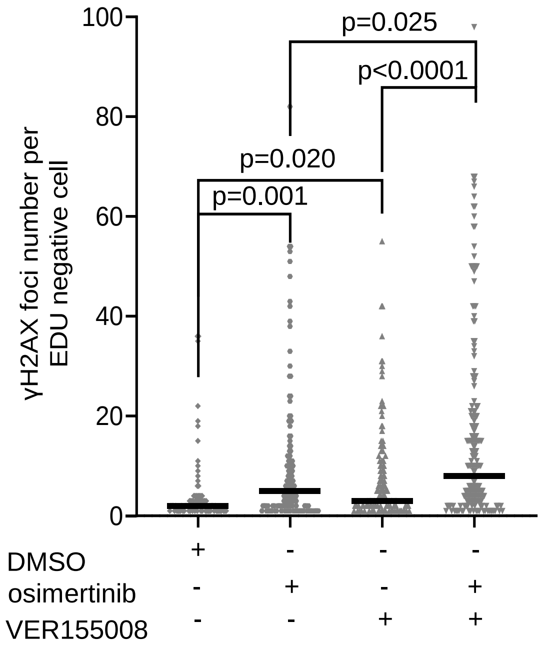

Supplementary Figure S13. DSB due to EGFR or HSP70 inhibition. Quantification of γH2AX foci in EdU negative HCC827 cells following treatment with HSP70 inhibitor VER155008 (5 µM) or EGFR inhibitor osimertinib (5 µM) alone or in combination for 2 hours. The p-value was calculated using Student's t-test.

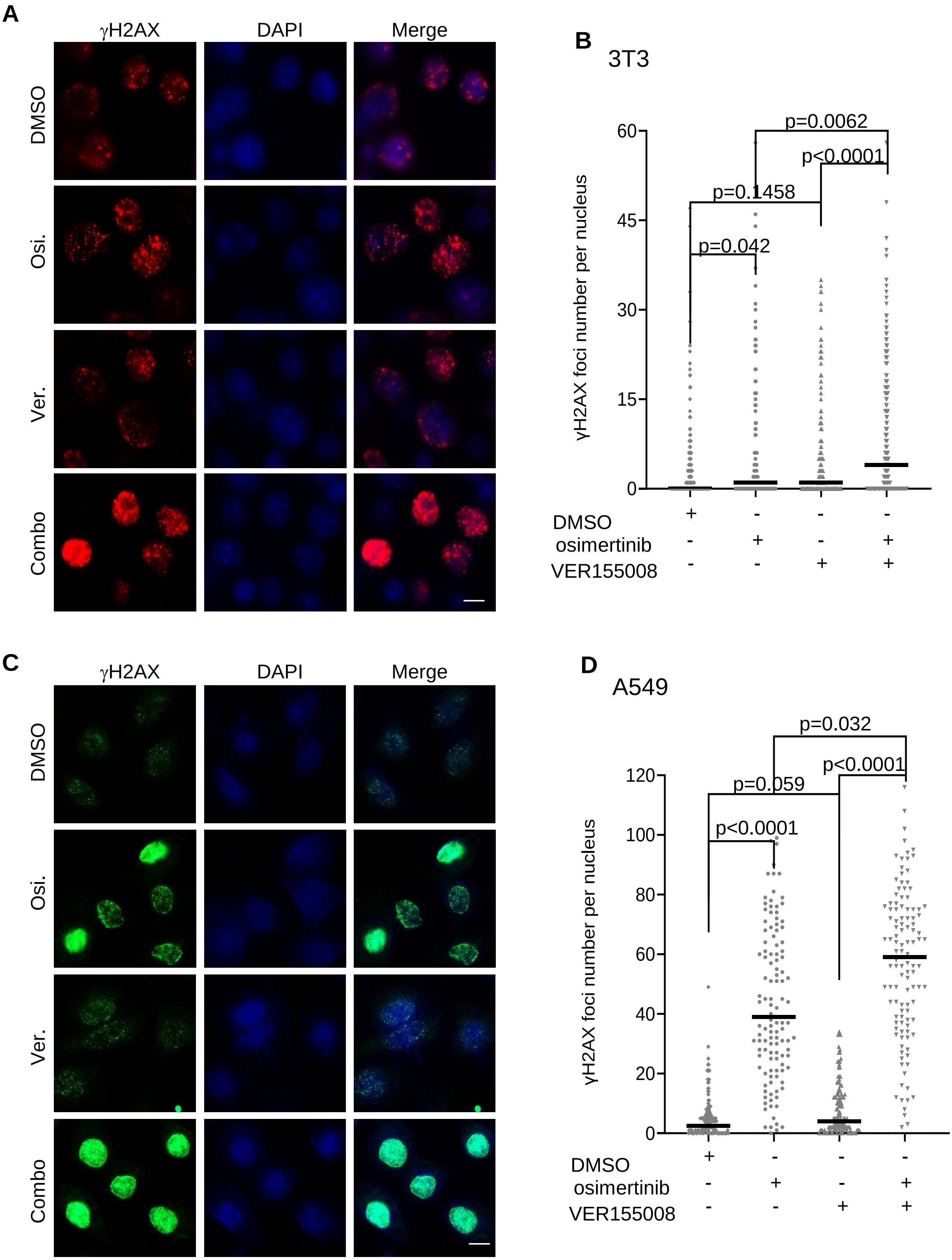

Supplementary Figure S14. DSB due to EGFR or HSP70 inhibition in 3T3 and A549 cells. (A) and (C) Representative images of IF staining of γH2AX in 3T3 and A549 cells following treatment with HSP70 inhibitor VER155008 (10 µM) or EGFR inhibitor osimertinib (3T3: 2.5 µM, A549: 10 µM) alone or in combination overnight. Nuclei were counterstained with DAPI (blue). Scale bar: 20 µm. (B) and (D) Quantification of γH2AX foci number using Image J software. The p-value was calculated using the student's t-test.

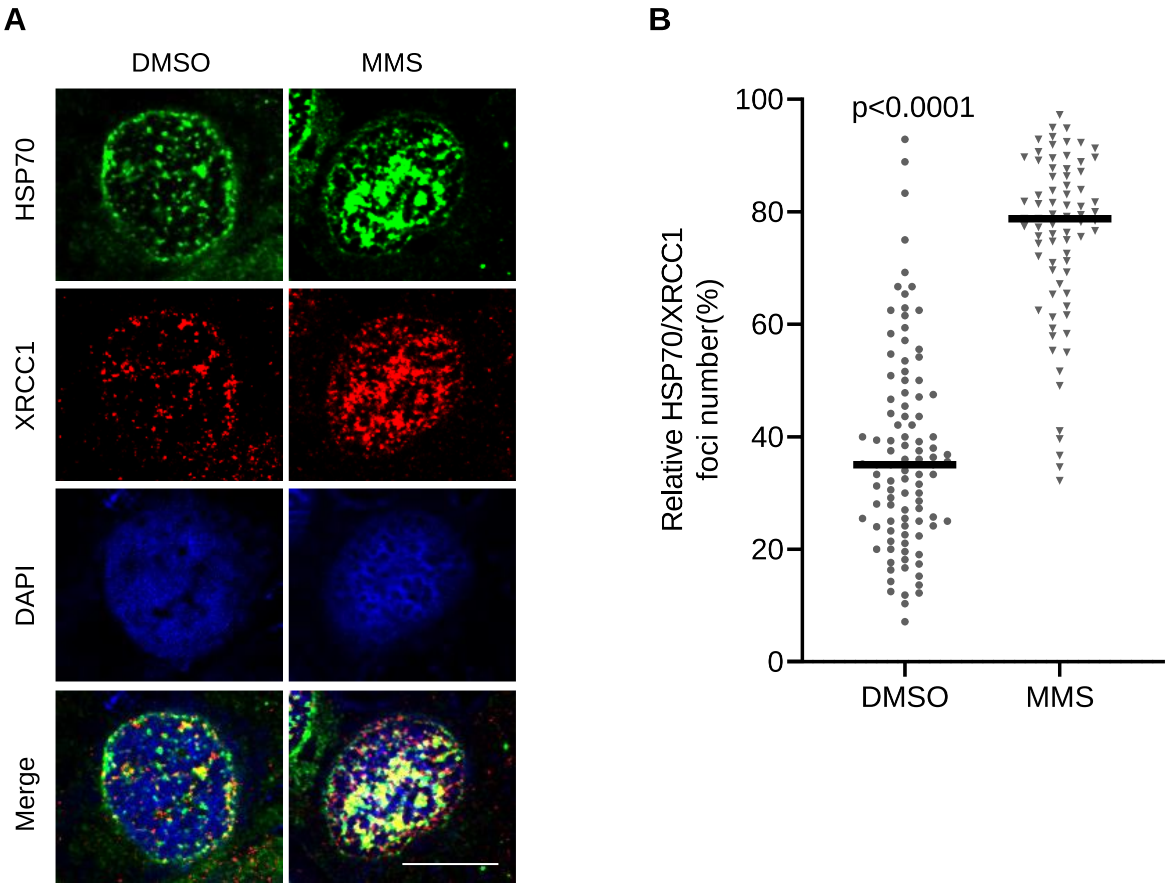

Supplementary Figure S15. Co-localization of HSP70 to XRCC1 foci (BER site). (A) Representative images shows HSP70 foci and XRCC1 foci with or without MMS (0.1%, 1 hour) treatment. Nuclei were counterstained with DAPI. Scale bar: 20 µm. (B) HSP70 foci that are co-localized with XRCC1 foci were quantified with the Image J software. The p value was calculated by using the student's t-test.

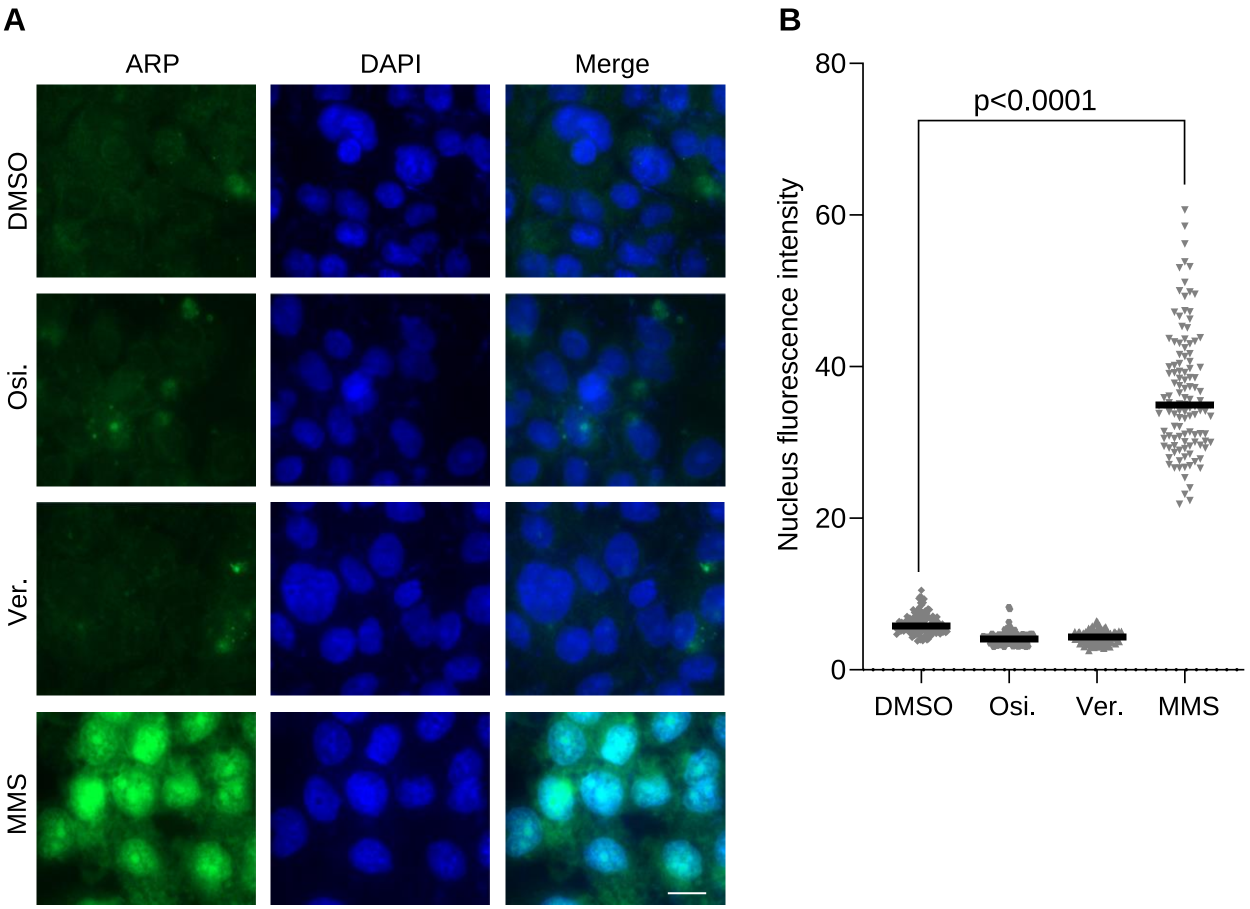

Supplementary Figure S16. Base damage due to EGFR or HSP70 inhibition. (A) IF staining of base damage in the cell using ARP. HCC827 cells were treated with or without VER155008 (5 µM, overnight) or EGFR inhibitor osimertinib (5 µM, overnight) or MMS (1%, 1 hour). Nuclei were counterstained with DAPI. Scale bar: 20 µm. (B) Quantification of ARP intensity per nucleus in each sample. The p-value was calculated using Student's t-test.

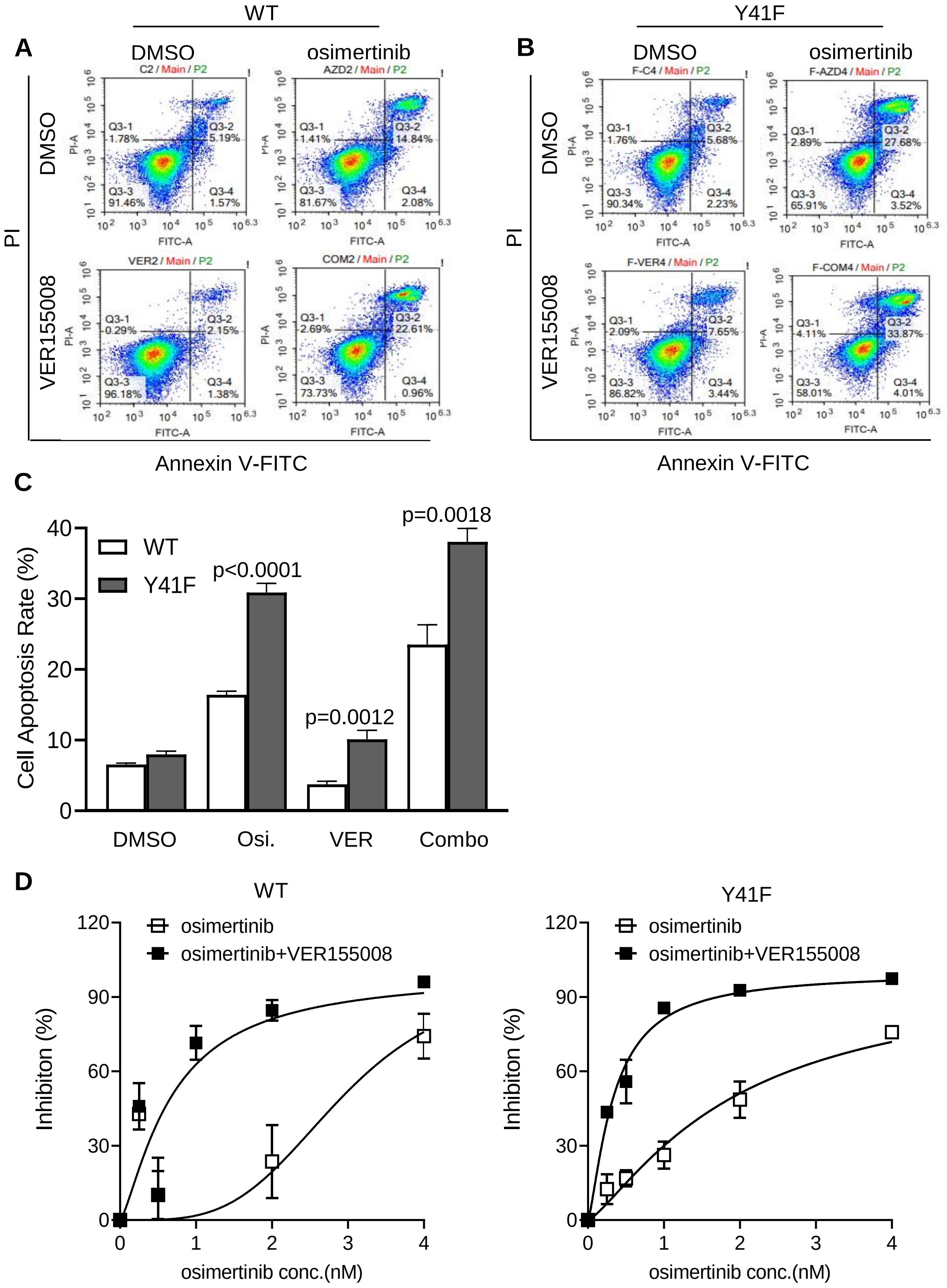

Supplementary Figure S17. HSP70 inhibitor VER155008 and EGFR-TKI osimertinib synergistically kill WT and Y41F HCC827 cells. (A) and (B) Flow cytometry-based apoptosis analysis of WT and Y41F HCC827 cells treated with DMSO (untreated control) or VER155008 (10 µM) and osimertinib (5 µM) individually or in combination for 24 hours. (C) Quantification of the percentage of apoptotic cells (Q2 in panel A) in different groups of WT, Y41F HCC827 cells. The p-value was calculated using the student's t-test. (D) The synergy between the HSP70 inhibitor VER155008 and the EGFR-TKI inhibitor osimertinib was assayed by clonogenic assay. The values are means ± SEM of three independent clonogenic assays. Panel D is the inhibition curve of varying concentrations of osimertinib from 0 to 4 nM in combination with VER155008 (0 or 1 µM).

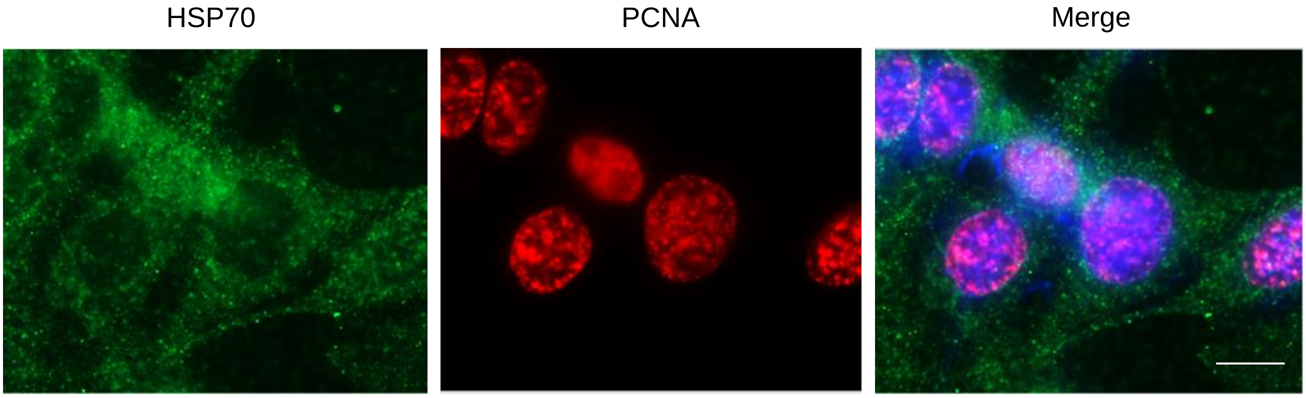

Supplementary Figure S18. IF staining of HSP70 (green) and PCNA (red) in MEF cells. Nuclei were counterstained with DAPI (blue). Scale bar: 20 µm.
